# Contribution to the knowledge of the Carabus (Archiplectes) satyrus Kurnakov, 1962, species complex in Abkhazia (Coleoptera, Carabidae, Carabini)

**DOI:** 10.3897/zookeys.463.8499

**Published:** 2014-12-12

**Authors:** Igor A. Solodovnikov, Alexandr S. Zamotajlov, Dmitriy D. Fominykh, Andrey Y. Titarenko

**Affiliations:** 1 Vitebsk State P.M. Masherov University, Moskovskyi prospect, 33, Vitebsk 210038, Belarus; 2 Kuban State Agrarian University, Kalinin St. 13, Krasnodar 350044, Russia; 3 Russian Entomological Society, Kuban Branch, Kalinin St. 13, Krasnodar 350044, Russia; 4 Publicly Traded Company “Morpho Absoloni”, Bolotnikovskaya St. 5/3, Moscow 117556, Russia

**Keywords:** Coleoptera, Carabidae, Carabus (Archiplectes) satyrus species complex, Abkhazia, taxonomy, distribution, new status, new subspecies

## Abstract

This study is based on a comparative analysis of extensive material of Carabus (Archiplectes) satyrus Kurnakov, 1962, its various forms and related taxa recently collected by the authors and some other collectors in Abkhazia. The status or specific affiliations of several subspecies are changed and a subspecies is described. Carabus (Archiplectes) besleticus Kurnakov, 1972, **stat. n.** is treated as a separate species housing six hitherto established subspecies in addition to the nominal type: Carabus (Archiplectes) besleticus mtsaranus Kurnakov, 1972, Carabus (Archiplectes) besleticus duripshensis Kurnakov, 1972, Carabus (Archiplectes) besleticus napraensis Belousov & Zamotajlov, 1993, Carabus (Archiplectes) besleticus dsychvensis Kurnakov, 1972, Carabus (Archiplectes) besleticus adzinbai Retezár, 2013, and Carabus (Archiplectes) besleticus resheviensis **subsp. n.** Carabus (Archiplectes) satyrus is treated as monotypical while the specific status of Carabus (Archiplectes) pseudopshuensis Zamotajlov, 1991, earlier proposed by Fominykh and Zamotajlov (2012), is confirmed based on the morphological and morphometric data.

## Introduction

Carabus (Archiplectes) satyrus Kurnakov, 1962, has been described from the vicinities of the settlement Gulripsh in the Gulripsh District of Abkhazia. Type material of the nominotypical subspecies originates from an interfluve of the rivers Kealasur and Kodor. Other subspecies of Carabus (Archiplectes) satyrus were hitherto known from the south slope of Bzybian Mountain Range, spreading from the orographic left bank of River Bzyb in the west to the right bank of River Kealasur in the east. One further subspecies, Carabus (Archiplectes) satyrus pseudopshuensis Zamotajlov, 1991, described from a single specimen, descends from the right bank of River Bzyb (environs of the Village Pskhu) in Abkhazia. Our study provides several informative morphological features: first of all the fully inflated endophallus preparations, structure of aggonoporius (= copulatory pieces), and some morphometric characters. It revealed that Carabus (Archiplectes) satyrus satyrus actually differs essentially from the other known subspecies. Based on these data, we change the specific affiliation of Carabus (Archiplectes) satyrus besleticus Kurnakov, 1972, Carabus (Archiplectes) satyrus duripshensis Kurnakov, 1972, Carabus (Archiplectes) satyrus mtsaranus Kurnakov, 1972, Carabus (Archiplectes) satyrus napraensis Belousov & Zamotajlov, 1993, Carabus (Archiplectes) satyrus adzinbai Retezár, 2013; the next paginal valid name, Carabus (Archiplectes) besleticus Kurnakov, 1972, stat. n. is hereafter applied. This species comprises all subspecies listed above. The shape of the fully inflated endophallus preparations, the structure of the aggonoporius, and the shape of the apical lamella of aedeagus of populations of *Archiplectes* collected at Mt. Dzykhva – type locality of Carabus (Archiplectes) satyrus dsychvensis Gottwald, 1985 – confirm that this taxon should be also attributed to Carabus (Archiplectes) besleticus, and thus its specific affiliation is changed to Carabus (Archiplectes) besleticus dsychvensis Gottwald, 1985. Material collected by the authors in environs of Village Pskhu, including the recently defined by Prof. Dr. A. Kazadaev type locality (pers. comm) of Carabus (Archiplectes) satyrus pseudopshuensis, appreciably differ in some structures (fully inflated endophallus preparations, aggonoporius and apical lamella of aedeagus) from the other taxa of the *Carabus satyrus* – *besleticus* complex. This allows us to confirm its specific status, recently proposed by Fominykh and Zamotajlov (2012). Material from a valley of one of the left tributaries of River Bzyb – River Reshevie – at the north slopes of Bzybian Mountain Range, originated from an altitude of 700 m and higher, proved the sympatry of two taxa related to Carabus (Archiplectes) satyrus. It displays the well-known pattern of the genus *Carabus*: the larger sized form occurs together with smaller one. Studies of the fully inflated endophallus preparations have also allowed us to establish reliable distinctions between these forms and interpret them as two separate species, the smaller form as Carabus (Archiplectes) pseudopshuensis Zamotajlov, and the larger one as a new subspecies Carabus (Archiplectes) besleticus resheviensis subsp. n. A distribution map of Carabus (Archiplectes) satyrus, Carabus (Archiplectes) besleticus and Carabus (Archiplectes) pseudopshuensis is given in Fig. 7.

Species of the complex resemble habitually forms of Carabus (Archiplectes) juenthneri Reitter, 1899. The latter species possesses very high morphological polymorphism in different features, this having resulted in description of its numerous forms and subspecies. However, they differ in having generally more robust than in the Carabus (Archiplectes) satyrus species complex body, larger, more transverse, and stronger cordate pronotum. Nevertheless, some populations of Carabus (Archiplectes) juenthneri from the right bank of river Aguripsta near village Pskhu are hardly distinguishable in habitus from Carabus (Archiplectes) pseudopshuensis and can be reliably recognized only by the shape of aedeagus and structure of endophallus and aggonoporius. Carabus (Archiplectes) juenthneri possesses larger aedeagus, apical lamella with two sharper hollows and more prominent tubercles laterally; preputial tubercle smaller, strongly sclerotized laterally and posteriorly, aggonoporius of different shape, much smaller than in Carabus (Archiplectes) besleticus and Carabus (Archiplectes) pseudopshuensis, with lobes being prominently dilated apically and rounded laterally, separated amidst by membranous folder.

Forms with elytral sculpture resembling Carabus (Archiplectes) reitteri reitteri Retowsky, 1885 (in particular, the type specimens of Carabus (Archiplectes) reitteri pshuensis Gottwald, 1985, and Carabus (Archiplectes) juenthneri acheicus Zamotajlov, 1991) are not considered in the present publication. According to I. Retezár’s personal communication as well as personal data of the authors, similar specimens are extremely rare in some local populations of both Carabus (Archiplectes) juenthneri Reitter, 1899, and Carabus (Archiplectes) pseudopshuensis Zamotajlov, 1991. Apparently, they should be interpreted as aberrations of the above-mentioned species or of their forms. Unquestionable identification of the taxa described as Carabus (Archiplectes) reitteri pshuensis and Carabus (Archiplectes) juenthneri acheicus seems to be impossible at present, since Carabus (Archiplectes) reitteri pshuensis Gottwald, 1985, has been described from the junction zone of geographic ranges (or hybridization zone) of Carabus (Archiplectes) juenthneri and Carabus (Archiplectes) pseudopshuensis, while the type locality of Carabus (Archiplectes) juenthneri acheicus is simply unknown, despite I. Retezár’s conclusion (2013).

## Material and methods

The following abbreviations are used for the depositories of the specimens examined:

cBAS Coll. A.S. Bondarenko (Krasnodar, Russia)

cFDD Coll. D.D. Fominykh (Krasnodar, Russia)

cKVM Coll. V.M. Kotsur (Vitebsk, Belarus)

cPIG Coll. I.G. Pljushtch (Kiev, Ukraine )

cPNYu Coll. N.Yu. Pichugin (Vladimir, Russia)

cPRYu Coll. R.Yu. Panin (Lviv, Ukraine)

cPSM Coll. S.M. Pavlyuchuk (Stavropol, Russia)

cSAA Coll. A.A. Safronov (Tula, Russia)

cSIA Coll. I.A. Solodovnikov (Vitebsk, Belarus)

cTAYu Coll. A.Yu. Titarenko (Moscow, Russia)

cZAM Coll. A.S. Zamotajlov (Krasnodar, Russia)

ZISP Zoological Institute of the Russian Academy of Sciences (St. Petersburg, Russia)

Measurements were taken as follows, with abbreviations: GBL – general body length, measured from the tips of mandibles to the elytral apex; SBL – standard body length, measured from the anterior margin of the clypeus to the elytral apex; HW – width of head, measured as the maximum linear distance across the head, including the compound eyes; PW – maximum width of pronotum measured at its broadest point; PB – minimum width of pronotum, measured at its narrowest point near the hind angles; PL – length of pronotum, measured along its median line; EL – length of elytra, measured from the basal border in the scutellar region to the apex of the sutural angle; EW – maximum width of elytra measured at their broadest point; PW/HW ratio; PW/PL ratio; PW/PB ratio; EW/PW ratio; EL/EW ratio. All these features and distribution of their values were analyzed in course of the discriminant analysis.

Both digital photographs of imago and drawings of the genitalia were prepared by the first author, general view photographs of the holotype of Carabus (Archiplectes) besleticus resheviensis by the third author, and three photographs (Figs 60, 61, 65) were copied from I. Retezár (2008). For the present study, we measured 391 specimens of *Carabus*. Graphic building was executed with the help of computer program STATISTICA (data analysis software system), StatSoft Inc., 2001 (version 6).

## Taxonomy

### Carabus (Archiplectes) satyrus

Taxon classificationAnimaliaColeopteraCarabidae

Kurnakov, 1962

Figs 1 , 2 , 7 , 8–11 , 34–41

Carabus (Neoplectes) satyrus Kurnakov, 1962: 33 (“Goulripch”).
Carabus (Archiplectes) satyrus satyrus : Gottwald 1985: 310.
Carabus (Archiplectes) satyrus satyrus : Bousquet et al. 2003: 132; Retezár 2008: 40.
Carabus (Tribax) satyrus satyrus Kurnakov: Deuve 2004: 274.

#### Comparative material examined.

86 specimens were examined (47 specimens measured, 15 male genitalia preparations studied): 1 male, Abkhazia, Gulripsh Distr., “Shervadshidzevskyi les” near Merkheul Village, 200 m, 17–20.V.2012, leg. D. Fominykh, A. Bondarenko (cFDD); 4 males, 4 females, Abkhazia, Gulripsh Distr., “Shervadshidzevskyi les” near Bagmarani Village, 200 m, 27.IV–25.VI.2013, leg. D. Fominykh, A. Bondarenko (cFDD); 3 males, 4 females, Abkhazia, Gulripsh Distr., NW slopes of Gurzul Mt. Range near Merkheul Village, 200 m, 27.IV–25.VI.2013, leg. D. Fominykh, A. Bondarenko (cFDD); 16 males, 31 females, Abkhazia, Gulripsh Distr., NW slopes of Gurzul Mt. Range near Merkheul Village, 170–230 m, left tributary of Machara River, hornbeam, alder, rhododendron (*Rhododendron ponticum*) forest site, 05.V–04.VII.2013, leg. I. Solodovnikov, S. Solodovnikova, V. Kotsur, S. Pavlyuchuk, N. Pichugin (cSIA, cFDD, cKVM, cPSM, cPNYu); 4 males, 5 females, Abkhazia, Gulripsh Distr., Gurzul Mt. Range near Merkheul Village, rivulet 1, left tributary of Machara River, hornbeam, alder, rhododendron (*Rhododendron ponticum*) forest site, 170 m, 43°59'17.91"N / 041°11'10.24"E, 11.V–08.VII.2014, leg. I. Solodovnikov, S. Solodovnikova, S. Pavlyuchuk (cSIA, cPSM); 6 males, 8 females, Abkhazia, Gulripsh Distr., left bank of River Kelasur, “Shervadshidzevskyi les”, gorge, hornbeam, alder, rhododendron (*Rhododendron ponticum*) forest site, 123–130 m, 42°58'54.43"N / 041°06'15.63"E, 11.V–08.VII.2014, leg. I. Solodovnikov, S. Solodovnikova, S. Pavlyuchuk (cSIA, cPSM).

#### Description.

Large form, males 31.1–35.7 (33.8) mm, females 32.2–39.8 (36.1) mm long, slender. Underside black, dorsum normally with bright metallic lustre, green, dark blue, bronze, crimson, violet, seldom black, with transitional color forms in males, females less nitidous, sometimes matte. Body shape is the narrowest within the studied species complex.

Head not inflated. Pronotum extremely variable in shape, from subquadrate to transverse, more often cordate, lateral sides with deep hollow before hind angles. PW/PL = 1.13–1.43 (1.32) in males and 1.03–1.57 (1.37) in females, hind angles strongly protruding backwards and sidewards, pointed apically. Median groove smooth to completely obliterated in some female specimens. Disk transversally rugose, with rugosity gradually strengthening towards median groove. Elytra strongly elongate to ovate (generally the most slender within studied species complex), more elongate in males, with inconspicuous depression in the middle in some female specimens. EL/EW = 1.64–2.02 (1.84) in males and 1.60–1.97 (1.80) in females. Elytral sculpture nearly identical in males and females, forming precise series of elongate links. The main morphometric measurements of the studied populations are presented in Table 2.

**Table 1.** Morphometric characteristic of males (n = 67) and females (n = 68) of Carabus (Archiplectes) pseudopshuensis.

Species/subspecies, locality, number of specimens studied	GBL*	SBL	HW	PW	PB	PL	EL	EW	PW/PL	PW/PB	EL/EW	EL/PL	EW/PW
**Males**													
*pseudopshuensis* vall. riv. Belaya, 1000 m, 1 ex	30.0	27.0	4.5	7.0	5.0	4.5	17.0	9.0	1.56	1.4	1.89	3.78	1.29
*pseudopshuensis* vall. riv. Byaul, 1100 m, 5 ex	29.0–32.0 30.6	26.0–29.0 27.6	4.5–5.5 4.84	6.5–7.5 6.84	5.5–6.0 5.74	5.0	16.5–18 17.36	9.0–10.0 9.56	1.3–1.5 1.37	1.17–1.25 1.19	1.78–1.86 1.82	3.3–3.6 3.47	1.33–1.49 1.4
*pseudopshuensis* Village Sanchara, 800 m, 5 ex	28.4–32.75 30.11	26.2–30.3 27.77	4.4–5.45 4.85	6.6–8.35 7.18	5.0–6.1 5.51	4.75–5.4 5.18	16–18.85 17.24	9.15–11.1 10.14	1.28–1.55 1.39	1.26–1.37 1.3	1.64–1.84 1.7	3.19–3.49 3.33	1.33–1.49 1.42
*pseudopshuensis* Village Bitaga (Pskhu), 700 m, 20 ex	26.45–32 29.47	24.25–30.0 27.22	4.25–5.0 4.69	5.9–7.2 6.7	4.4–5.8 5.24	4.5–5.5 5.09	14.75–18 16.7	8.8–11 9.87	1.2–1.41 1.32	1.09–1.4 1.28	1.51–1.86 1.69	2.91–3.5 3.28	1.34–1.66 1.48
*pseudopshuensis* vall. riv. Reshevie, 700 m, 20 ex	29.0–32.0 30.39	26.55–30.0 28.22	4.4–6.0 5.13	6.35–8.2 7.17	4.85–7.0 5.75	4.9–5.5 5.16	16.5–22.7 17.84	9.2–12.4 10.6	1.21–1.64 1.39	1–1.41 1.25	1.45–2.17 1.69	3.09–4.63 3.47	1.33–1.6 1.48
*pseudopshuensis* Dou Pass, 1300 m, 1 ex	31.0	29.0	5.0	7.0	6.0	6.0	17.0	10.4	1.17	1.17	1.63	2.83	1.49
*pseudopshuensis* vall. riv. Bzyb, 900 m, 5 ex	29.0–31.0 30.3	27.0–29.5 28.4	5.0	6.5–7.0 6.86	5.0–6.5 5.84	5.0–6.0 5.46	17.0–18.0 17.56	10.0–11.0 10.32	1.17–1.4 1.26	1.08–1.3 1.18	1.59–1.8 1.7	3–3.56 3.23	1.43–1.69 1.51
*pseudopshuensis* Village Serebryanyi, 600 m, 10 ex	28.0–31.0 29.4	26.0–29.0 27.45	4.8–6.0 5.28	6.5–8.0 7.21	5.0–7.0 6.11	5.2–5.7 5.43	16.0–18.0 17.5	9.6–11.6 10.58	1.18–1.51 1.33	1–1.33 1.19	1.38–1.88 1.66	2.91–3.4 3.22	1.37–1.55 1.47
**Females**													
*pseudopshuensis* vall. riv. Belaya, 1000 m, 1 ex	31.0	29.0	5.0	7.5	5.5	5.0	17.0	8.0	1.5	1.36	2.13	3.4	1.07
*pseudopshuensis* vall. riv. Byaul, 1100 m, 5 ex	31.0–33.0 32.26	28.5–31.0 30.1	4.5–5.5 5.1	7.0–8.0 7.4	5.0–7.8 6.16	4.5–5.5 5.0	15.3–19.0 16.96	9.0–11.0 10.08	1.4–1.56 1.48	1.03–1.4 1.23	1.47–1.89 1.69	3.06–3.78 3.4	1.29–1.43 1.36
*pseudopshuensis* Village Sanchara, 800 m, 4 ex	31.0–32.2 31.63	29.0–29.65 29.26	4.8–5.55 5.15	7.0–8.0 7.58	5.85–6.5 6.11	5.3–5.0 5.33	18.25–19.0 18.53	10.45–10.8 10.56	1.32–1.56 1.42	1.2–1.28 1.24	1.69–1.81 1.75	3.34–3.8 3.49	1.31–1.49 1.4
*pseudopshuensis* Village Bitaga (Pskhu), 700 m, 20 ex	29.55–33 31.99	27.75–30.5 29.46	4.65–5.35 5.01	7.0–8.5 7.42	5.35–6.5 5.98	5.0–5.8 5.4	16.8–19.4 17.98	9.6–10.95 10.25	1.21–1.55 1.38	1.08–1.42 1.24	1.6–1.98 1.76	3.08–3.59 3.33	1.13–1.51 1.39
*pseudopshuensis* vall. riv. Reshevie, 700 m, 20 ex	29.0–34.0 31.58	27.0–32.0 29.13	4.5–5.45 4.92	6.5–8.55 7.4	5.2–6.8 6.04	4.75–6.3 5.25	16.5–19.4 18.19	9.0–11.25 10.4	1.25–1.63 1.42	1.1–1.35 1.23	1.59–1.9 1.75	3.02–3.8 3.48	1.3–1.62 1.41
*pseudopshuensis* Dou Pass, 1300 m, 3 ex	31.5–33.0 32.17	29.5–31.0 30.17	5.0	7.3–7.8 7.53	6.0–6.3 6.1	5.0–5.3 5.17	17.5–19.0 18	9.6–11.6 10.53	1.38–1.5 1.46	1.22–1.25 1.23	1.64–1.82 1.71	3.5–3.58 3.48	1.28–1.59 1.4
*pseudopshuensis* vall. riv. Bzyb, 900 m, 5 ex	30.0–33.0 31.5	27.5–30.5 29.2	5.2–5.5 5.4	7.0–7.5 7.14	6.0	5.0–6.0 5.36	17.0–18.0 17.7	10.0–12.0 11.08	1.25–1.4 1.34	1.17–1.25 1.19	1.5–1.7 1.6	3.0–3.6 3.32	1.43–1.63 1.55
*pseudopshuensis* Village Serebryanyi, 600 m, 10 ex	28.5–32.0 30.3	26.5–30.0 28.3	4.5–5.8 5.22	5.8–8.0 6.8	5.0–7.0 5.9	5.0–6.5 5.58	16.5–19.5 17.75	9.6–11.0 10.32	0.89–1.44 1.23	1.07–1.2 1.16	1.63–1.84 1.72	2.83–3.45 3.19	1.28–1.83 1.53

* for abbreviations see text.

**Table 2.** Morphometric characteristic of males (n = 16) and females (n = 31) of Carabus (Archiplectes) satyrus and males (n = 83) and females (n = 91) of Carabus (Archiplectes) besleticus subspecies.

Species/subspecies, locality, number of specimens studied	GBL*	SBL	HW	PW	PB	PL	EL	EW	PW/PL	PW/PB	EL/EW	EL/PL	EW/PW
**Males**													
*satyrus* Village Merkheul, 120–230 m, 16 ex.	31.1–35.7 33.84	28.6–33.1 31.22	5.0–5.65 5.39	6.8–8.6 7.84	5.75–6.85 6.33	5.65–6.6 5.97	18.2–21.4 19.75	9.95–11.35 10.73	1.13–1.43 1.32	1.06–1.36 1.24	1.64–2.02 1.84	3.03–3.68 3.32	1.22–1.49 1.37
*besleticus* *resheviensis* vall. riv. Reshevie, 700 m, 29 ex.	34.5–39.0 37.62	32.6–37.0 35.15	5.75–6.35 6.03	8.0–9.5 8.58	6.8–8.2 7.19	5.5–7.0 6.27	21–22.7 21.45	12.1–15 13.51	1.18–1.55 1.37	1.1–1.3 1.19	1.42–1.78 1.59	3–3.79 3.43	1.41–1.83 1.58
*besleticus* *adzinbai* Mt. Akibakhu, 2000 m, 1 ex.	31.0	29.0	6.5	7.5	6.5	6.0	20.0	12.4	1.25	1.15	1.61	3.33	1.65
*besleticus* *mtsaranus* vall. riv. Mtsara, 550–750 m, 10 ex.	35.25–37.1 35.77	31.8–34.0 32.82	5.5–5.75 5.59	7.5–8.85 8.19	6.4–7.2 6.81	5.55–6.9 6.1	19.6–22.15 20.79	11.15–12.55 11.87	1.14–1.48 1.35	1.15–1.26 1.2	1.65–1.85 1.75	2.84–3.87 3.42	1.3–1.63 1.45
*besleticus* *besleticus* Mt. Birtzkha, 300 m, 20 ex.	32.0–38.0 34.68	30.5–34.65 32.29	5.4–6.3 5.87	7.55–9.75 8.36	6.25–8.0 7.21	5.65–6.5 6.12	20–22.5 21.34	10.9–13 12.23	1.26–1.53 1.37	1.03–1.33 1.17	1.62–1.86 1.75	3.29–3.75 3.49	1.23–1.63 1.47
*besleticus* *dsykhvensis* Mt. Dzykhva, 2000 m, 19 ex.	23.3–26.5 25.2	21.7–25.4 23.41	4.0–5.0 4.46	5.65–6.4 6.13	4.6–5.6 5.06	4.0–4.6 4.29	13.4–15.8 14.56	8.0–9.2 8.65	1.2–1.58 1.43	1.07–1.37 1.22	1.56–1.85 1.68	3.08–3.59 3.39	1.29–1.51 1.41
*besleticus* *duripshensis* Bzybian Mt. Range. 420–550 m, 2 ex.	32.45–32.5 32.48	29.75–30.0 29.88	4.75–5.3 5.03	7.2–7.6 7.4	5.65–5.75 5.7	5.15–5.85 5.5	19.1 19.1	10.6–10.8 10.7	1.30–1.40 1.35	1.27–1.32 1.30	1.77–1.80 1.79	3.26–3.71 3.49	1.42–1.47 1.45
*besleticus* *napraensis* NW of Bzybian Mt. Range, 1900–2000 m, 2 ex.	28.8–30.7 29.75	26.45–28.15 27.3	4.85–5.15 5.0	6.65–6.85 6.75	5.25–5.8 5.53	5.25–5.25 5.25	17.0–17.65 17.33	9.8–10.55 10.18	1.27–1.31 1.29	1.18–1.27 1.22	1.67–1.74 1.70	3.24–3.36 3.30	1.48–1.54 1.51
**Females**													
*satyrus* Village Merkheul, 120–230 m, 31 ex.	32.2–39.75 36.11	29.5–37.25 33.24	5.25–6.3 5.81	7.5–9.5 8.58	6.0–7.6 6.9	5.35–8.0 6.33	18.0–24.15 21.04	10.9–13.4 11.71	1.03–1.57 1.37	1.08–1.36 1.24	1.6–1.97 1.80	2.48–3.79 3.35	1.22–1.51 1.37
*besleticus* *resheviensis* vall. riv. Reshevie, 700 m, 28 ex	37.15–43 40.61	34.15–41 37.9	5.7–7.2 6.6	8.25–10 9.43	6.5–9.0 7.92	5.6–7.2 6.5	19.8–25 23.59	12.25–16 13.74	1.29–1.67 1.45	1.06–1.33 1.2	1.52–1.92 1.72	2.96–4.08 3.64	1.3–1.67 1.46
*besleticus* *adzinbai* Mt. Akibakhu, 2000 m, 10 ex.	32–35.75 33.33	30.0–32.9 31.24	5.85–6.5 6.06	7.5–9.5 8.33	6.5–8.5 7.31	6.0–7.5 6.66	20.65–22.5 21.7	11.25–13 12.65	1.14–1.42 1.25	1.0–1.26 1.14	1.64–1.84 1.72	2.93–3.67 3.27	1.32–1.73 1.53
*besleticus* *mtsaranus* vall. riv. Mtsara, 550–750 m, 10 ex.	37.5–41.25 39.28	34.5–37.75 36.11	5.8–6.25 6.01	8.3–9.5 9.12	7.0–8.15 7.43	5.85–7.0 6.51	21.7–24 22.74	12.1–13.5 12.67	1.26–1.56 1.41	1.13–1.32 1.23	1.73–1.98 1.8	3.29–3.86 3.5	1.29–1.47 1.39
*besleticus* *besleticus* Mt. Birtzkha, 300 m, 20 ex.	38.0–40.75 38.95	35.15–38.0 36.45	5.85–6.5 6.27	8.5–11.0 9.74	7.0–9.0 8.19	6.0–7.2 6.68	22.45–24.0 23.22	12.15–14.4 13.06	1.25–1.62 1.46	1.11–1.31 1.19	1.64–1.86 1.78	3.19–3.88 3.48	1.13–1.47 1.35
*besleticus* *besleticus* Village Abzhakva, 70 m, 1 ex.	39.0	36.0	6.5	10.3	8.1	7.0	22.5	13.5	1.46	1.27	1.67	3.21	1.32
*besleticus* *dsykhvensis* Mt. Dzykhva, 2000 m, 18 ex.	23.3–26.5 25.2	23.65–26.3 25.18	4.0–4.9 4.44	6.0–7.3 6.47	5.0–6.8 5.53	4.2–4.9 4.44	14.65–17.0 15.5	8.65–10 9.38	1.3–1.62 1.46	1.03–1.3 1.17	1.5–1.79 1.66	3.33–3.67 3.49	1.34–1.59 1.45
*besleticus* *duripshensis* Bzybian Mt. Range, 420–550 m, 2 ex.	32.5–32.6 32.55	30.0–30.3 30.15	5.25–5.25 5.25	7.25–7.3 7.28	5.75–6.3 6.03	5.65–5.75 5.7	19.0–20.0 19.5	10.5–10.75 10.63	1.27–1.28 1.28	1.16–1.26 1.21	1.81–1.86 1.83	3.36–3.48 3.42	1.45–1.47 1.46
*besleticus* *napraensis* NW of Bzybian Mt. Range, 1900–2000 m, 2 ex.	31.7–33.7 32.7	29.35–30.6 29.98	5.35–5.5 5.43	7.6–7.9 7.75	6.35–6.45 6.4	5.15–5.3 5.23	19.0–20.35 19.68	10.5–12.0 11.25	1.48–1.49 1.48	1.20–1.22 1.21	1.70–1.81 1.75	3.69–3.84 3.76	1.38–1.52 1.45

* for abbreviations see text.

Lamella of aedeagus short, of triangular form. Structure of endophallus testifies to the assignment of this form to the *reitteri*-group (since the previous authors did not properly analyze this morphological structure, it lead them to an erroneous interpretation of the subspecific structure of this species). Saccellus short, of rectangular shape, resembling saccellus of Carabus (Archiplectes) juenthneri, with S-shaped fold on the right, paracellar lobe rounded, protruding laterally, endophallus with asymmetric position of inner structures (when viewed frontally). Ostium lobe small, sometimes faintly developed. Aggonoporius small.

#### Differential diagnosis.

**Figure 1.**
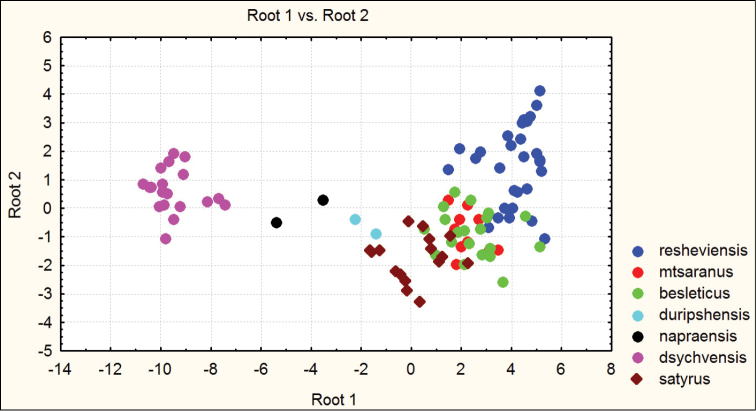
Distribution of morphometric characters in males of Carabus (Archiplectes) besleticus and Carabus (Archiplectes) satyrus subspecies constructed using discriminant analysis based on 8 parameters.

**Figure 2.**
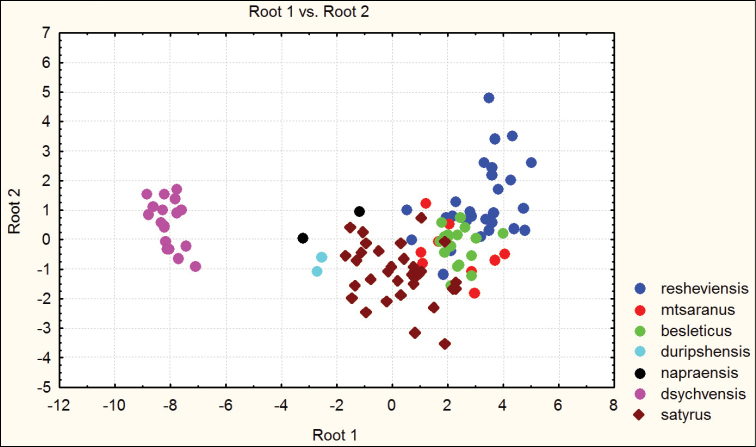
Distribution of morphometric characters in females of Carabus (Archiplectes) besleticus and Carabus (Archiplectes) satyrus subspecies constructed using discriminant analysis based on 8 parameters.

Strongly resembles Carabus (Archiplectes) besleticus besleticus from which it differs in a more elongate and less ovate body shape, the more precise elytral sculpture, a more convex pronotum with sharper hind angles, a very narrow base of the pronotum (PW/PB being the highest in the species complex in question), and also by the structure of the male genitalia. For the illustration of morphometric characters of this species, see Figures 1 and 2.

#### Distribution.

Populates low mountain forest belts in an interfluve of the r. Kodor and Kelasur, from altitude of 50 m up to 500 m.

#### Habitat.

Prefers forests of the Colchic (= Colchian) type admixed with beech, chestnut, and rhododendron (*Rhododendron ponticum*). Activity of imago proceeds from April until July. Carabus (Archiplectes) koltzei koltzei Rost, 1889, Carabus (Megodontus) septemcarinatus Motschulsky, 1840, and Carabus (Sphodristocarabus) armeniacus laevilineatus Ganglbauer, 1887, also occur together with this species (being much more abundant).

### Carabus (Archiplectes) besleticus

Taxon classificationAnimaliaColeopteraCarabidae

Kurnakov, 1972 stat. n.

Figs 1 , 2 , 3 , 4 , 5 , 6 , 7 , 16–21 , 22–27 , 28–33 , 34–41 , 42–49 , 50–57 , 58–61 , 62–65 , 66–73 , 74–77

#### Remarks.

The structure of the endophallus testifies to the attribution of this form to the *reitteri* group. Saccellus large, conical, smoothly rounded apically, with faintly developed or missing S-shaped fold on the right, paracellar lobe very faintly developed, endophallus with rather symmetric position of inner structures (when viewed frontally). Ostium lobe large, of dulled conical shape. Lamella of aedeagus elongated, with prominent hollow on the right before apex. Aggonoporius large, strongly sclerotized. This type of endophallus is characteristic for all subspecies described subsequently, earlier attributed to Carabus (Archiplectes) satyrus. Based on such pronounced differences in the structure of the endophallus from that of Carabus (Archiplectes) satyrus, it is proposed to raise the status of Carabus (Archiplectes) besleticus from subspecies to species.

**Figure 3.**
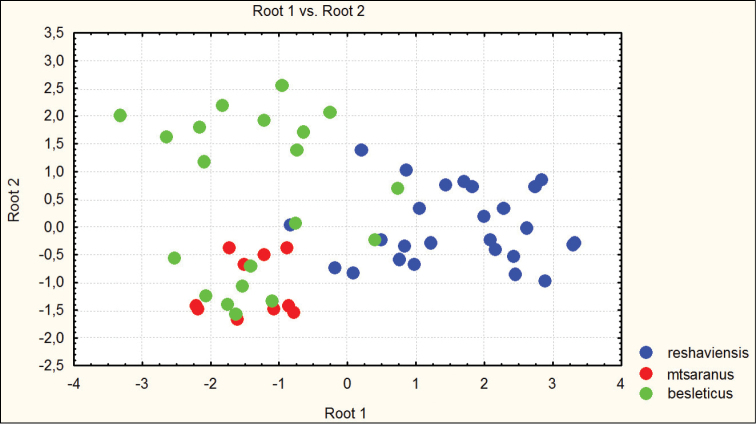
Distribution of morphometric characters in males of Carabus (Archiplectes) besleticus subspecies constructed using discriminant analysis based on 8 parameters.

**Figure 4.**
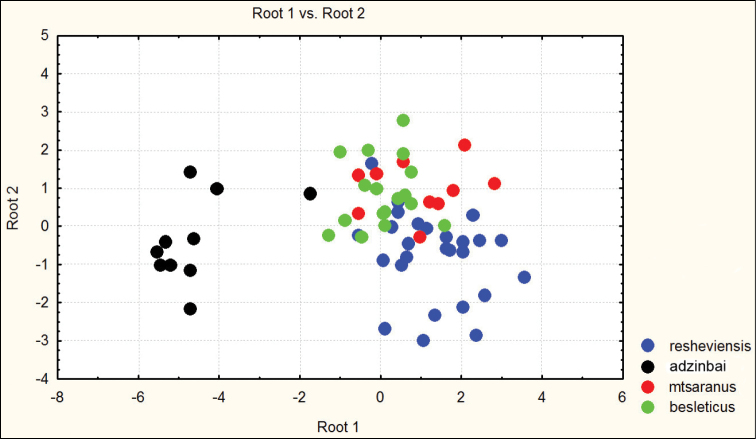
Distribution of morphometric characters in females of Carabus (Archiplectes) besleticus subspecies constructed using discriminant analysis based on 8 parameters.

**Figure 5.**
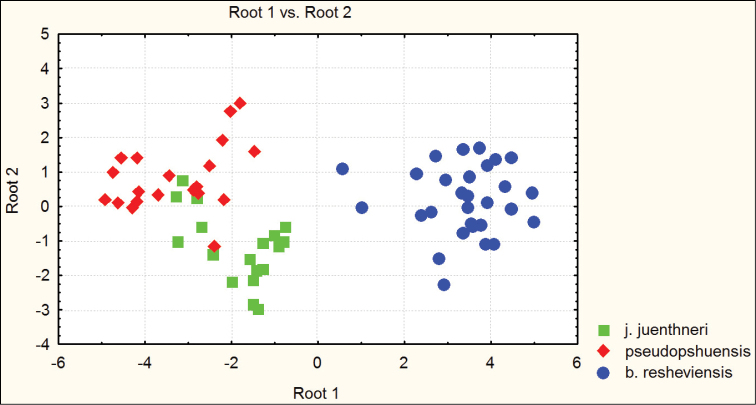
Distribution of morphometric characters in males of Carabus (Archiplectes) besleticus resheviensis (River Reshevie), Carabus (Archiplectes) pseudopshuensis (River Reshevie), and Carabus (Archiplectes) juenthneri juenthneri (Village Pskhu env.) constructed using discriminant analysis based on 8 parameters.

**Figure 6.**
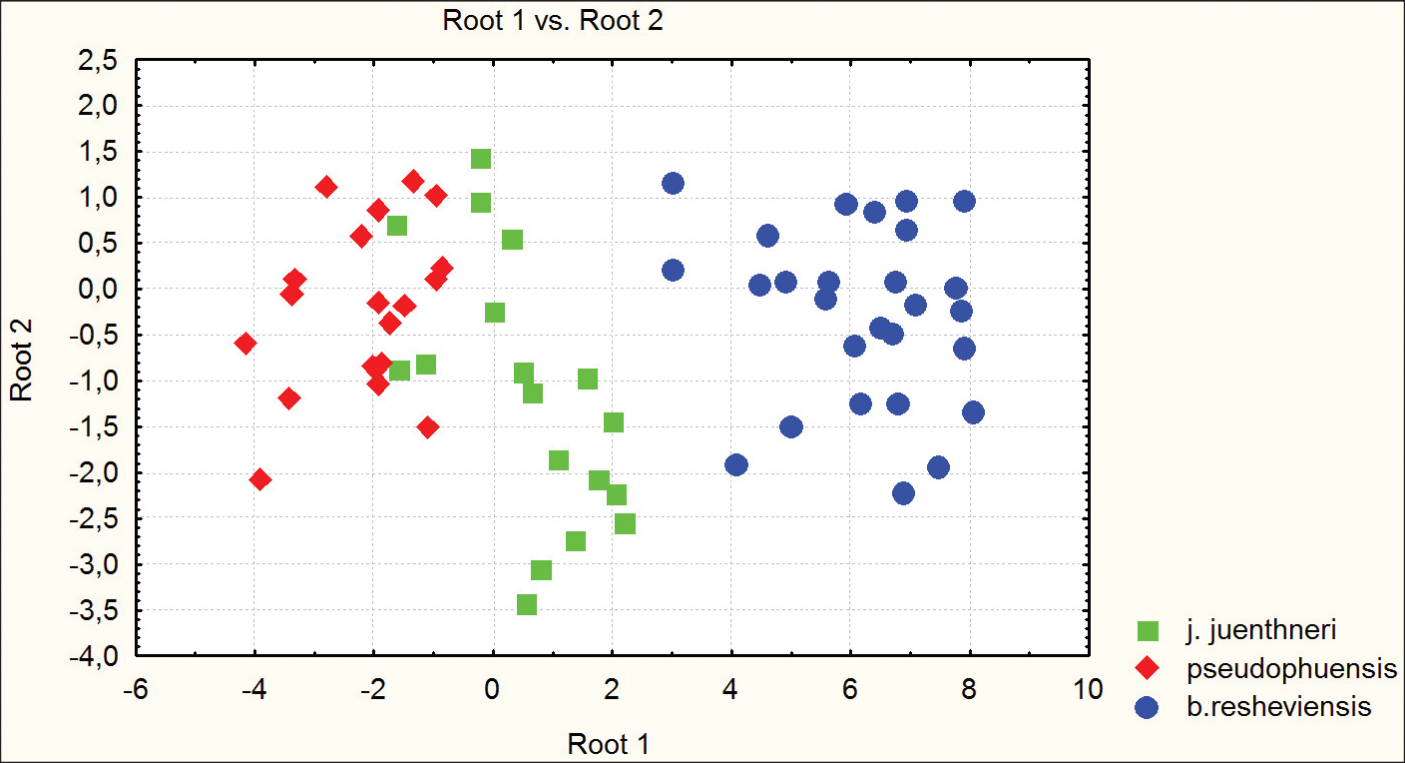
Distribution of morphometric characters in females of Carabus (Archiplectes) besleticus resheviensis (River Reshevie), Carabus (Archiplectes) pseudopshuensis (River Reshevie), and Carabus (Archiplectes) juenthneri juenthneri (Village Pskhu env.) constructed using discriminant analysis based on 8 parameters.

**Figure 7.**
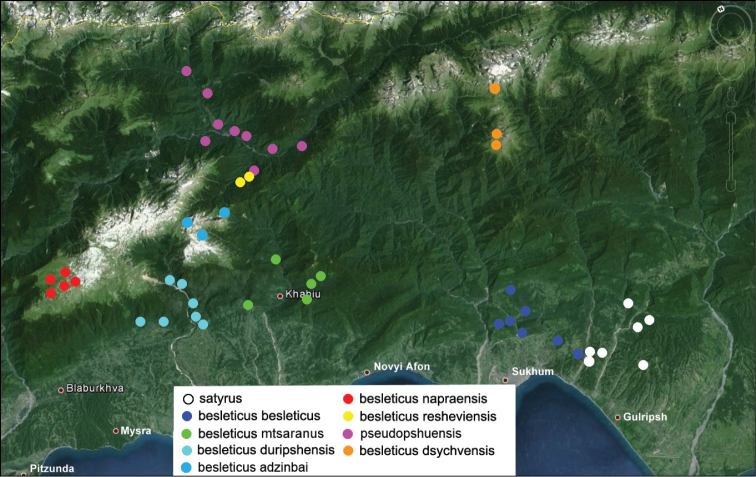
Distribution map of Carabus (Archiplectes) satyrus, Carabus (Archiplectes) besleticus, and Carabus (Archiplectes) pseudopshuensis in Abkhazia.

**Figures 8–15.**
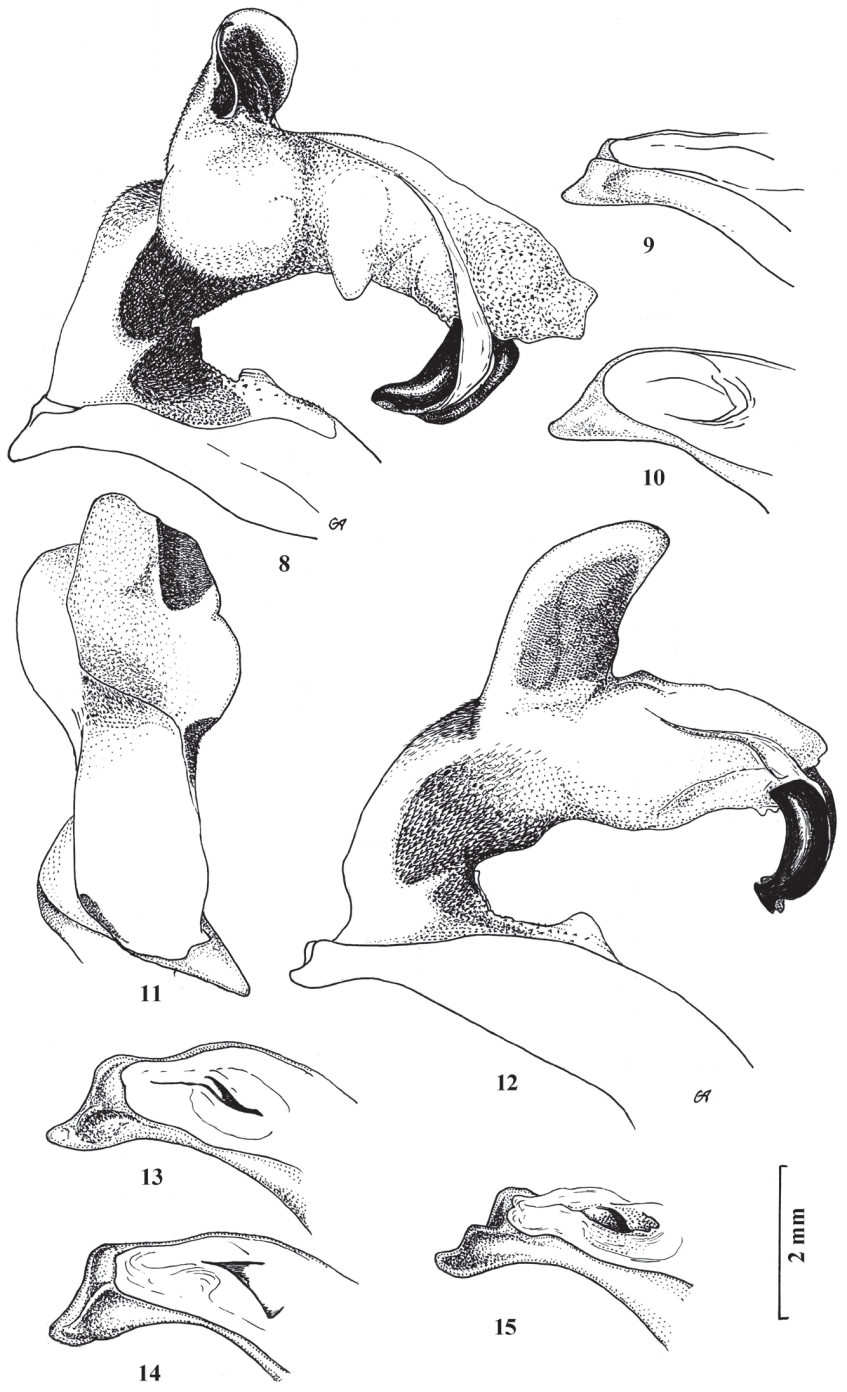
Fully inflated endophallus preparation and apical lamella of aedeagus of Carabus (Archiplectes) satyrus and Carabus (Archiplectes) pseudopshuensis. **8–11** Carabus (Archiplectes) satyrus **12–15** Carabus (Archiplectes) pseudopshuensis.

**Figures 16–21.**
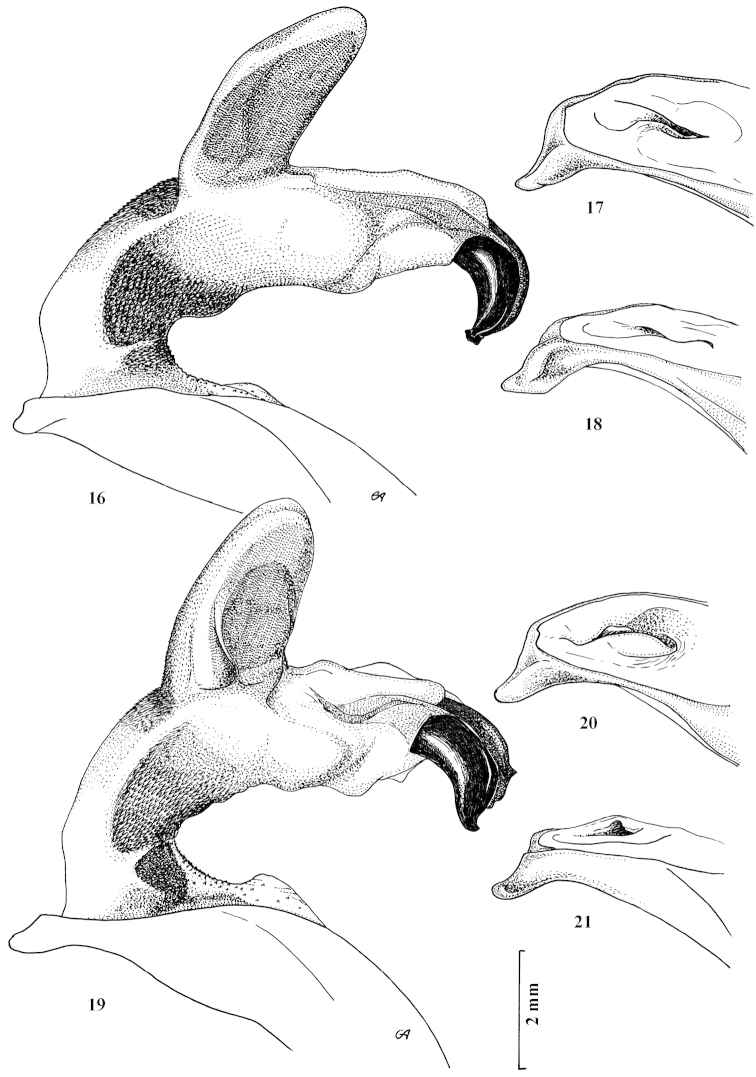
Fully inflated endophallus preparation and apical lamella of aedeagus of Carabus (Archiplectes) besleticus besleticus and Carabus (Archiplectes) besleticus mtsaranus. **16–18** Carabus (Archiplectes) besleticus besleticus **19–21** Carabus (Archiplectes) besleticus mtsaranus.

**Figures 22–27.**
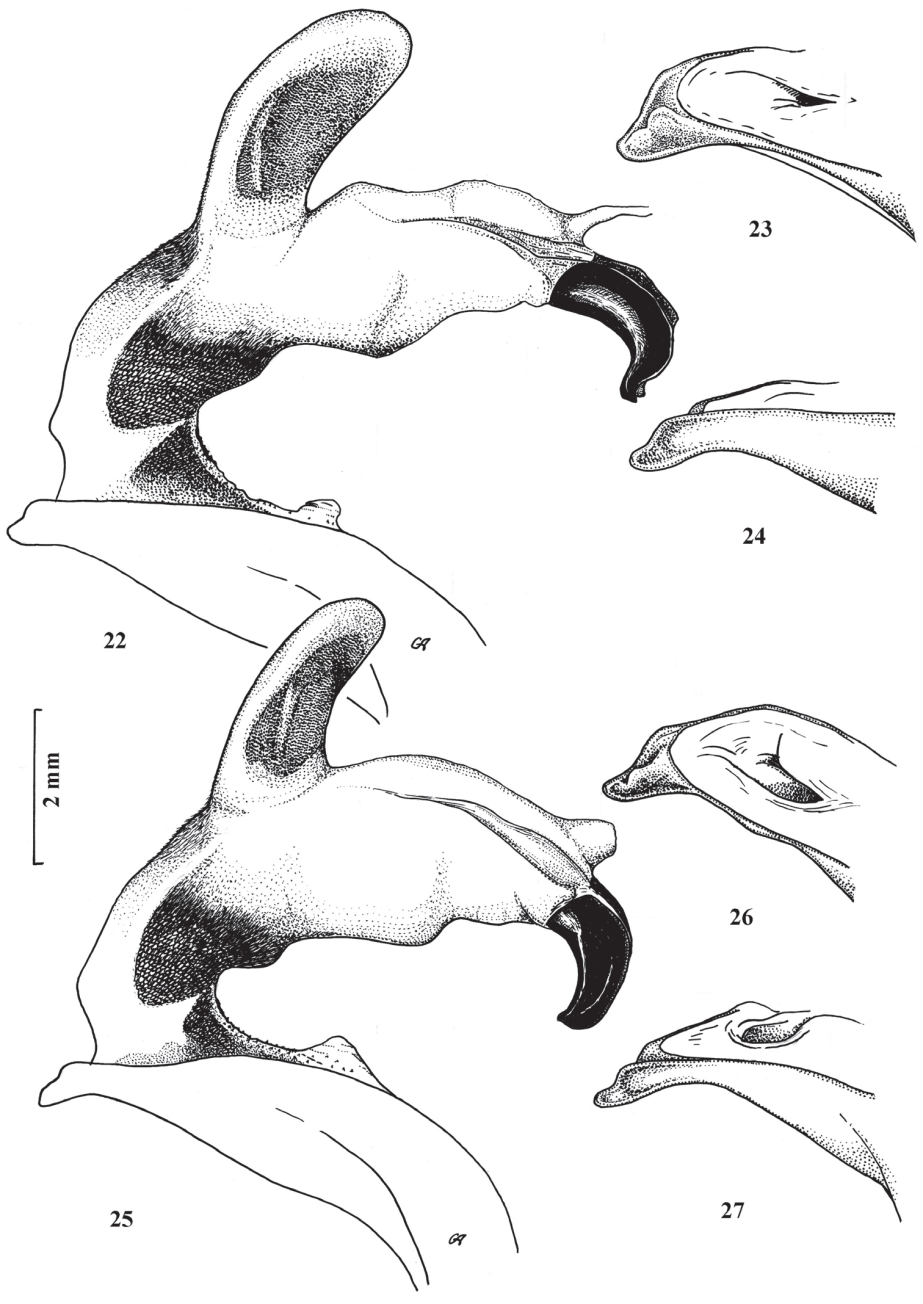
Fully inflated endophallus preparation and apical lamella of aedeagus of Carabus (Archiplectes) besleticus duripshensis and Carabus (Archiplectes) besleticus napraensis. **22–24** Carabus (Archiplectes) besleticus duripshensis **25–27** Carabus (Archiplectes) besleticus napraensis.

**Figures 28–33.**
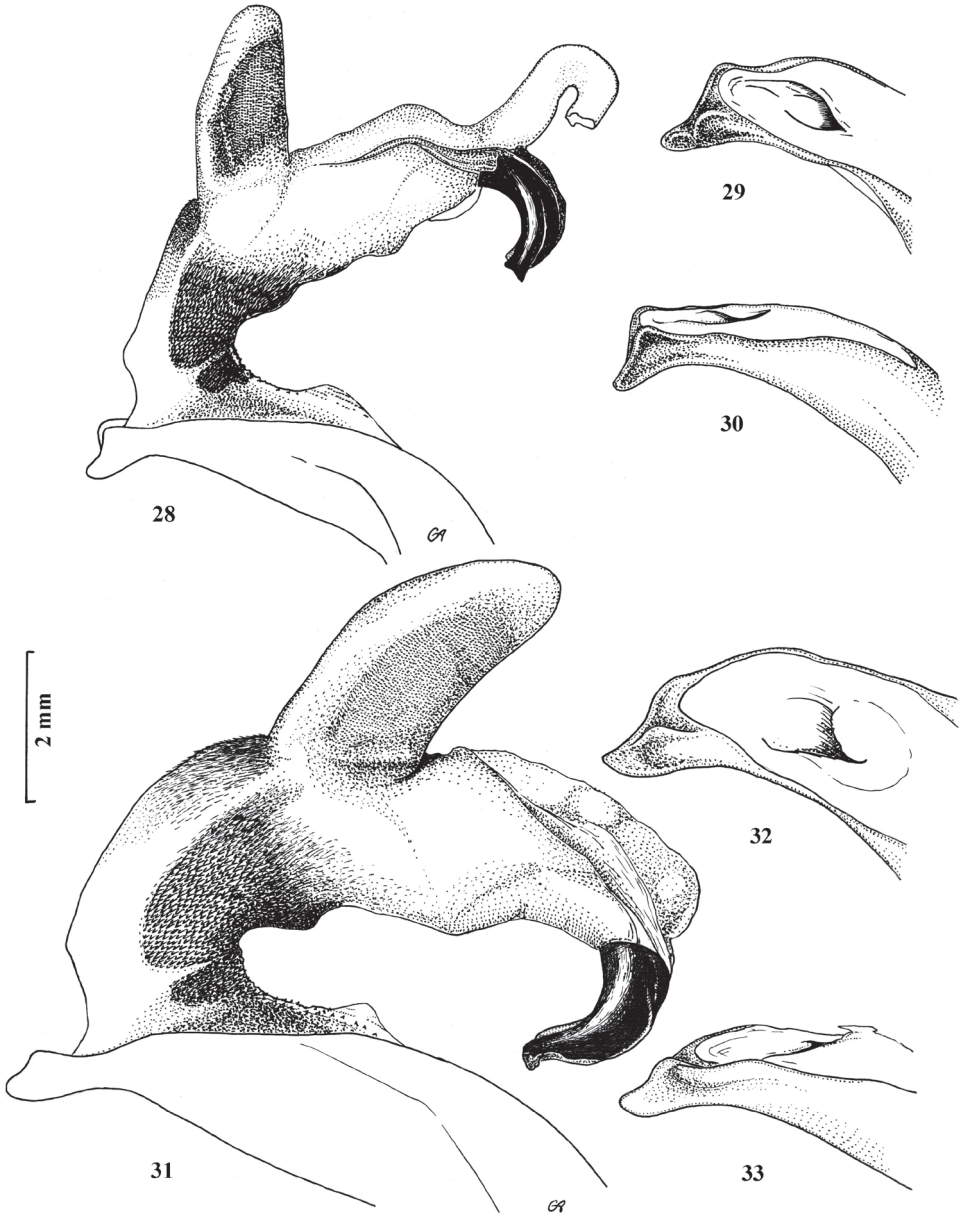
Fully inflated endophallus preparation and apical lamella of aedeagus of Carabus (Archiplectes) besleticus dsychvensis and Carabus (Archiplectes) besleticus resheviensis **28–30** Carabus (Archiplectes) besleticus dsychvensis **31–33** Carabus (Archiplectes) besleticus resheviensis.

**Figures 34–41.**
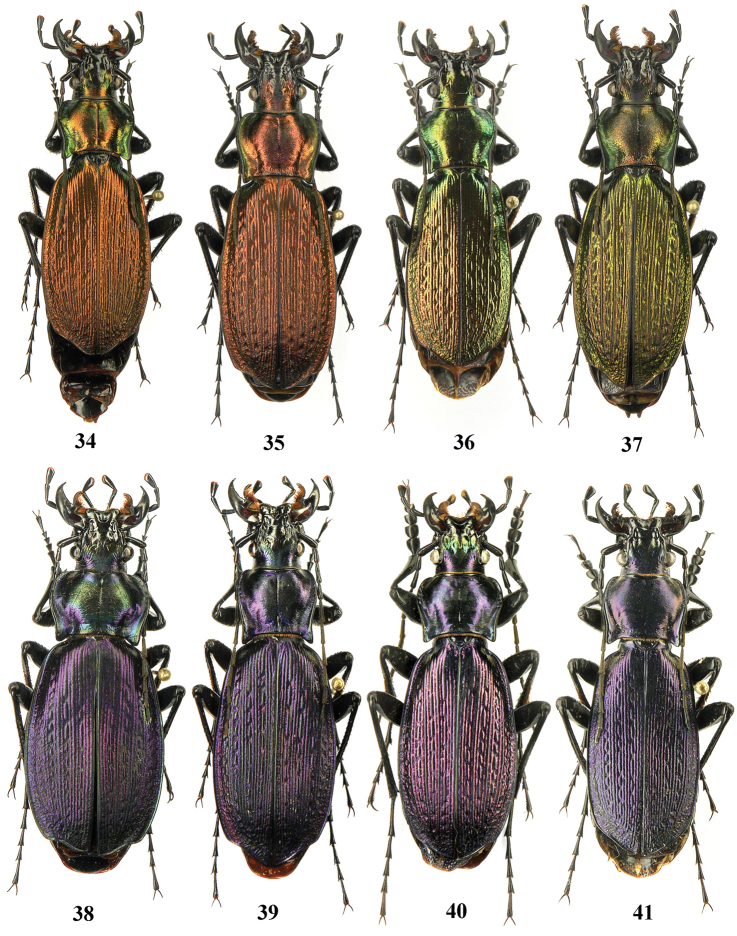
Dorsal habitus, Carabus (Archiplectes) satyrus, Abkhazia, Village Merkheul env.

**Figures 42–49.**
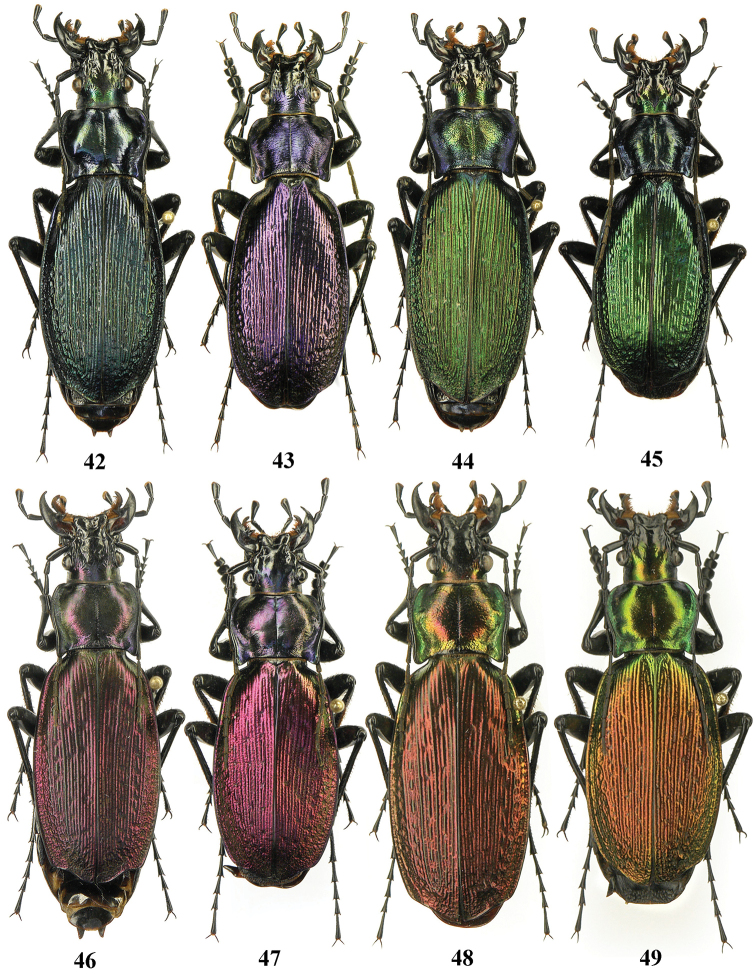
Dorsal habitus, Carabus (Archiplectes) besleticus besleticus, Abkhazia, Mt. Birtzkha.

**Figures 50–57.**
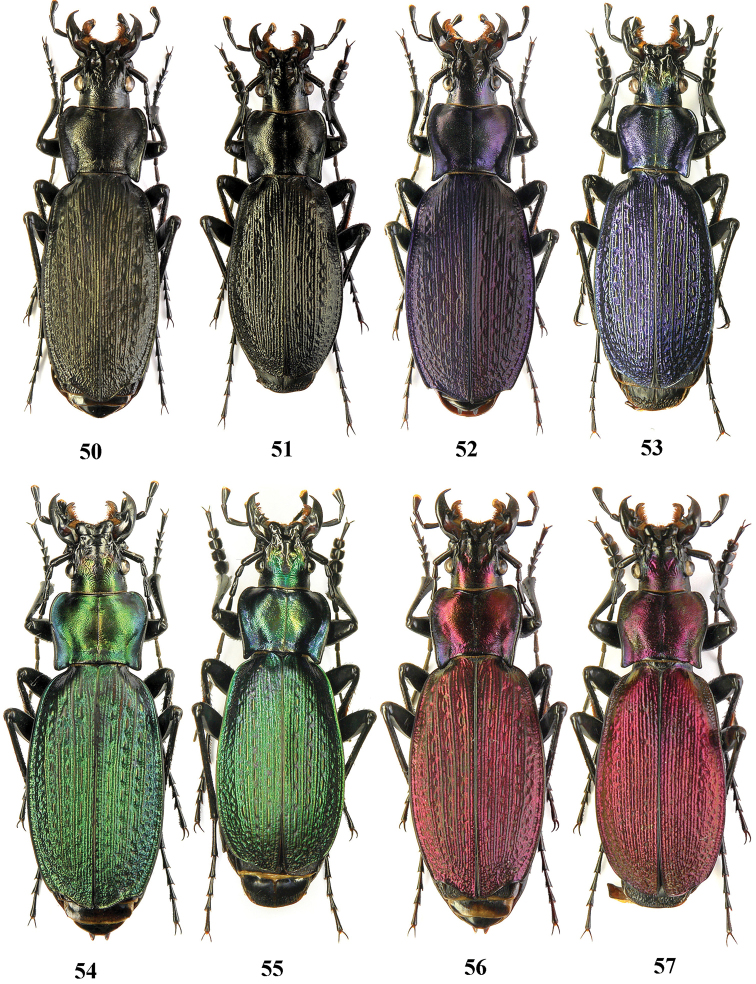
Dorsal habitus, Carabus (Archiplectes) besleticus mtsaranus, Abkhazia, Zashirbara Mt. Range, valley of River Mtsara.

**Figures 58–61.**
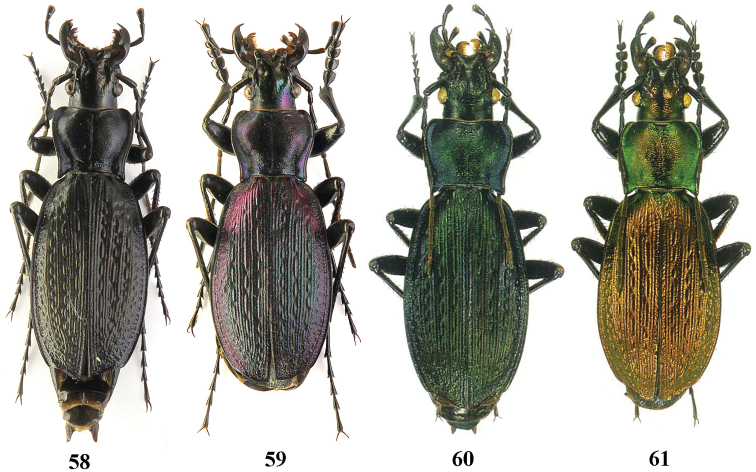
Dorsal habitus, Carabus (Archiplectes) besleticus duripshensis. **58–59** Abkhazia, S slope of Bsybian Mt. Range, valley of River Khipsta **60–61** Abkhazia, Bsybian Mt. Range, Duriph env. (after Retezár 2008).

**Figures 62–65.**
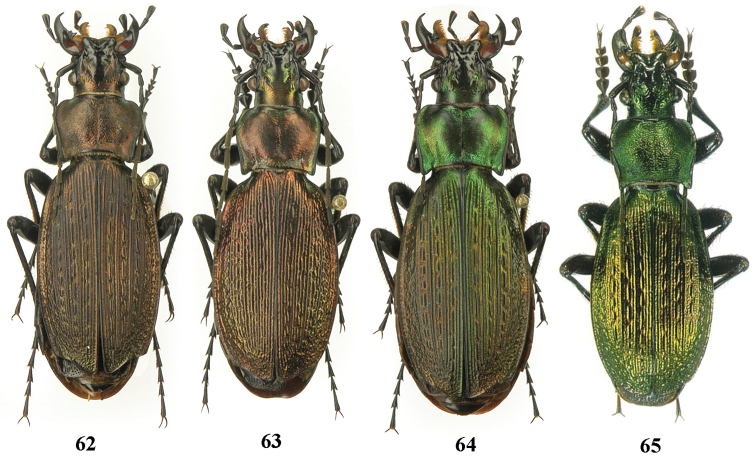
Dorsal habitus, Carabus (Archiplectes) besleticus napraensis. **62–64** Abkhazia, Bzybian Mt. Range, Mt. Chibzharga (paratypes) **65** same locality (paratype) (after Retezár 2008).

**Figures 66–73.**
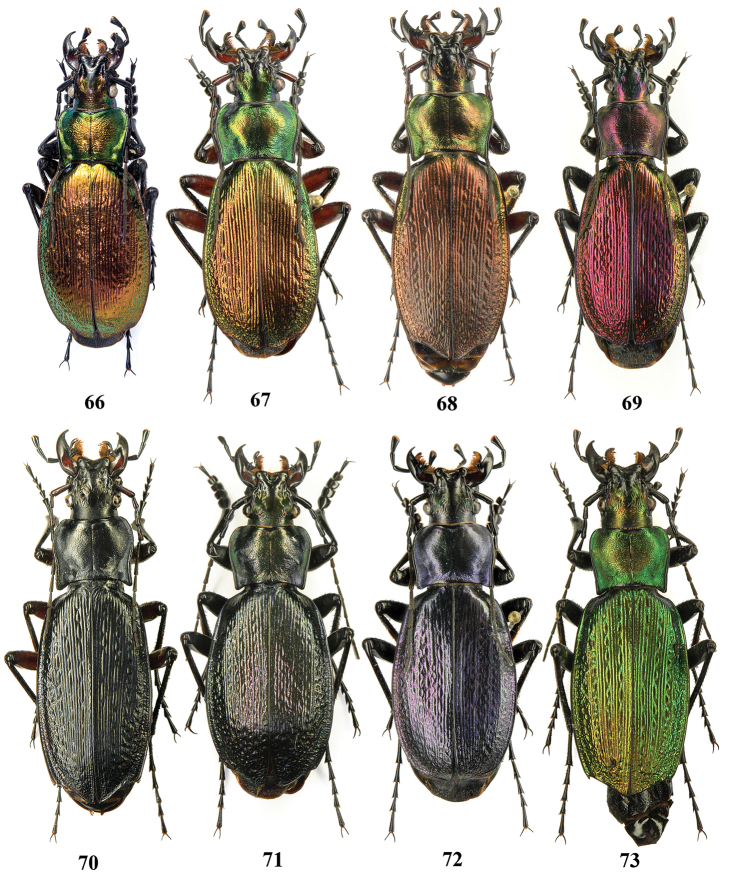
Dorsal habitus, Carabus (Archiplectes) besleticus resheviensis. **66** Abkhazia, N slopes of Bzybian Mt. Range, right bank of River Reshevie (holotype) **67–73** same locality (paratypes).

**Figures 74–77.**
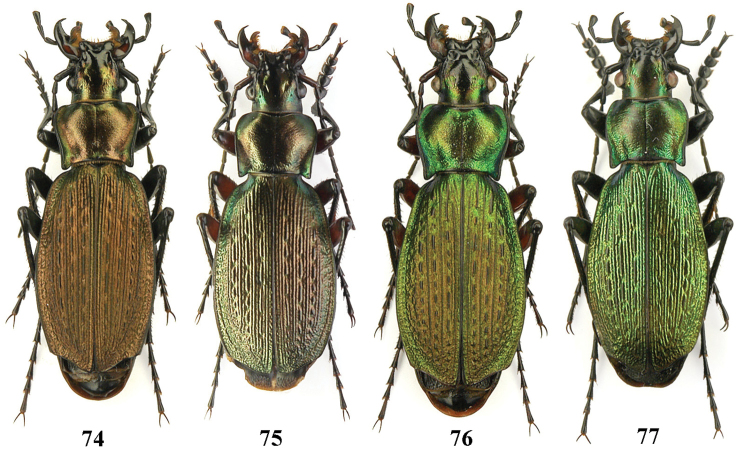
Dorsal habitus, Carabus (Archiplectes) besleticus dsychvensis, Abkhazia, Bzybian Mt. Range, SW slopes of Mt. Dzykhva.

### Carabus (Archiplectes) besleticus besleticus

Taxon classificationAnimaliaColeopteraCarabidae

Kurnakov, 1972

Figs 1 , 2 , 3 , 4 , 7 , 16–18 , 42–49

Carabus (Neoplectes) satyrus besleticus Kurnakov, 1972: 112 (“Vallée de la Besléta”).
Neoplectes reitteri gaskoi Kenyeri, 1975: 113.
Carabus (Archiplectes) satyrus besleticus : Gottwald 1985: 310; Bousquet et al. 2003: 132; Retezár 2008: 40.
Carabus (Tribax) satyrus besleticus : Deuve 2004: 274.

#### Comparative material examined.

176 specimens were examined (41 specimens measured, 11 male genitalia preparations studied): 59 males, 73 females, Abkhazia, Sukhum Distr. near Sukhum city, N slope of Mt. Birtzkha, 200 m, 26.IV–25.VI.2013, leg. D. Fominykh, A. Bondarenko (cFDD, cBAS, cSIA, cKVM); 1 female, Abkhazia, Sukhum Distr., vicinities of Village Abzhakva, left bank of River Basla, 80–90 m, box and beech forest site, 05–16.V.2013, leg. I. Solodovnikov, S. Solodovnikova, V. Kotsur, N. Pichugin (cSIA); 1 male, Abkhazia, Sukhum Distr., vicinities of Village Kveno-Linda, N slope of Mt. Birtskha, 120–140 m, box and beech forest site, N43°02'49.81", E041°04'01.23", 05–13.05.2012, leg. I. Solodovnikov (cSIA); 11 males, 10 females, Abkhazia, Sukhum Distr., vicinities of Village Kaman, N slope of Mt. Birtskha, 160 m, beech, linden, box, elder forest site, 05.V-04.VII.2013, leg. I. Solodovnikov, S. Solodovnikova, V. Kotsur, S. Pavlyuchuk, N. Pichugin (cSIA, cKVM, cPSM, cPNYu); 4 males, 12 females, Abkhazia, Sukhum Distr., right bank of River Kelasur, E slope of Mt. Anykhapaara, beech, alder, chestnut forest site, 135 m, 42°59'30.67"N / 041°05'59.66"E, 11.V–08.VII.2014, leg. I. Solodovnikov, S. Solodovnikova, S. Pavlyuchuk (cSIA, cPSM); 2 males, 3 females, Abkhazia, Sukhum Distr., vicinities of Village Kaman, N slope of Mt. Birtskha, right bank of River Basla, beech, linden, box, elder forest site, 160 m, 43°02'57.59"N / 041°03'48.67"E, 11.V–08.VII.2014, leg. I. Solodovnikov, S. Solodovnikova, S. Pavlyuchuk (cSIA, cPSM).

#### Description.

Large form, males 32.0–38.0 (34.7) mm, females 38.0–40.75 (39.0) mm long, robust. Underside black, dorsum normally with bright metallic luster, various colours of green, dark blue, bronze, crimson, violet, seldom black, with transitional color forms in males, females matte. Body massive, less slender than in Carabus (Archiplectes) satyrus.

Head not inflated. Pronotum of variable shape, from subcordate to transverse, lateral sides not to faintly sinuated. PW/PL = 1.26–1.53 (1.37) in males and 1.25–1.62 (1.46) in females, base rather broad, PW/PB = 1.03–1.33 (1.17) in males and 1.11–1.31 (1.19) in females, hind angles distinctly protruding backwards and faintly sidewards, rounded apically. Median groove distinct, microsculpture forming transversal rugosity, gradually strengthening towards median groove. Elytra strongly elongate to ovate, more elongate in males, with inconspicuous depression in the middle in both males and females. EL/EW = 1.62–1.86 (1.75) in males and 1.64–1.86 (1.78) in females. Elytral sculpture nearly identical in males and females, forming faint and smoothed series of elongated links. The main morphometric measurements of the studied populations are given in Table 2.

Lamella of aedeagus elongate, with a prominent hollow on the right side before the apex. Saccellus large, conical, smoothly rounded apically, paracellar lobe hardly developed, endophallus with symmetrical position of inner structures (when viewed frontally). Ostium lobe large, of blunted conical shape.

#### Differential diagnosis.

Habitually strongly resembles Carabus (Archiplectes) satyrus from which it differs in the less elongate and more rounded body shape, the different shape of pronotum, smoothed elytral sculpture with longer links, the different shapes of pronotum and male genitalia. The morphometric characters of this subspecies are illustrated in Figures 1–4.

#### Distribution.

Populates a low mountain forest belt in an interfluve of the River Gumista – East Gumista and Kelasur.

#### Habitat.

Prefers beech and beech-chestnut forest sites admixed with box, rich in ground litter on karstic landforms at altitudes from 50 to 500 m. Activity of imago proceeds from April until July. Carabus (Archiplectes) apollo phoebus Kurnakov, 1962, Carabus (Megodontus) septemcarinatus, Carabus (Sphodristocarabus) armeniacus rugatus Breuning, 1934 (natio *novotnyorum* Mandl, 1975), and Carabus (Procerus) caucasicus colchicus Motschulsky, 1844, occur together with this subspecies.

### Carabus (Archiplectes) besleticus mtsaranus

Taxon classificationAnimaliaColeopteraCarabidae

Kurnakov, 1972

Figs 1 , 2 , 3 , 4 , 7 , 19–21 , 50–57

Carabus (Neoplectes) satyrus mtsaranus Kurnakov, 1972: 114 (“Chaîne Bzybienne près du village de Mtsara”).
Carabus (Archiplectes) satyrus mtsaranus : Gottwald 1985: 310; Bousquet et al. 2003: 132; Retezár 2008: 41.
Carabus (Tribax) satyrus mtsaranus : Deuve 2004: 274.

#### Comparative material examined.

134 specimens were examined (31 specimens measured, 18 male genitalia preparations studied): 12 males, 17 females Abkhazia, Gudauta Distr., valley of River Aapsta, 700 m, V–VII.1993, leg. A. Zamotajlov, F. Miroshnikov (cZAM, cFDD); 49 males, 56 females, Abkhazia, Gudauta Distr, vicinities of Mtsara (Chiryuta), Zashirbara Mt. Range, source of River Mtsara, beech, blackberry, fern site, 575-690 m, 11.V-05.VII.2013, leg. I. Solodovnikov, S. Solodovnikova, V. Kotsur, N. Pichugin (cSIA, cKVM, cPSM, cPNYu, cFDD).

#### Description.

Large form, males 35.3–37.1 (35.8) mm, females 37.5–41.3 (39.3) mm long, robust. Underside black, dorsum normally with bright metallic lustre, dark blue, violet, black-violet, black, rather seldom green, bronze, crimson, females matte. Body massive.

Head not inflated. Pronotum of variable shape, from subcordate to cordate, transverse. PW/PL = 1.14–1.48 (1.35) in males and 1.26–1.56 (1.41) in females, with base narrower than in the nominotypical subspecies, PW/PB = 1.15–1.26 (1.20) in males and 1.13–1.32 (1.23) in females, hind angles strongly protruding backwards and sidewards, seldom only backwards, pointed apically. Median groove distinct, disk transversally or irregularly rugose, with rugosity gradually strengthening towards median groove. Elytra strongly elongate to ovate, more elongate in males, with quite inconspicuous depression in the middle in females and seldom in males. EL/EW = 1.65–1.85 (1.75) in males and 1.73–1.98 (1.80) in females. Elytral sculpture identical in males and females, forming coarse and precise series of short links. Table 2 lists the main morphometric measurements of the studied populations.

The shape of the male genitalia is practically identical to that of the other Carabus (Archiplectes) besleticus taxa. The endophallus differs from the one in Carabus (Archiplectes) satyrus, mainly in the shape of the dorsal appendix, which is more elongate and possesses a more extended form, characteristic for populations dwelling in an interfluve of r. Kealasur and Bzyb.

#### Differential diagnosis.

Resembles Carabus (Archiplectes) besleticus besleticus from which it differs in a more elongate and less ovate body shape, the different form of the hind angles of the pronotum, and in having a more coarse elytral sculpture with precise series of rather short links. The color of dorsum is usually more dull and darker than in the nominotypical and other subspecies. For the morphometric characters of this subspecies see Figures 1–4.

#### Distribution.

Populates southern slopes of Bzybian Mt. Range at an altitude of 400–1500 m in an interfluve of r. Gumista – Western Gumista and Khipsta.

#### Habitat.

Prefers beech and beech-chestnut forest sites, sometimes admixed with box, rich in ground litter at karstic landforms (sometimes even populating also quite sharp and steep ones). This subspecies also occurs at alpestrine and alpine meadows. Activity of imago, depending upon the altitude of the habitation, proceeds from April to August. Carabus (Archiplectes) apollo tenebricosus Kurnakov, 1962, Carabus (Microplectes) argonautarum reischitzi Mandl, 1955, Carabus (Tribax) apschuanus apschuanus Rost, 1893, Carabus (Tribax) constantinovi otcharensis Kurnakov, 1970. Carabus (Megodontus) septemcarinatus, Carabus (Sphodristocarabus) armeniacus dvorschaki Mandl, 1975, and Carabus (Procerus) caucasicus colchicus occur together with this subspecies.

### Carabus (Archiplectes) besleticus duripshensis

Taxon classificationAnimaliaColeopteraCarabidae

Kurnakov, 1972

Figs 1 , 2 , 7 , 22–24 , 58–61

Carabus (Neoplectes) satyrus duripshensis Kurnakov, 1972: 114 (“Chaîne Bzybienne près du village de Douripch”)
Carabus (Archiplectes) satyrus duripshensis : Gottwald 1985: 310; Bousquet et al. 2003: 132; Retezár 2008: 40.
Carabus (Tribax) satyrus duripshensis : Deuve 2004: 275.

#### Comparative material examined.

69 specimens were examined (4 specimens measured, 2 male genitalia preparations studied): 15 male, 18 female, Abkhazia, Gudauta Distr. near Village Khuap, V–VII.1993, leg. A. Zamotajlov, A. Miroshnikov (cZAM, cFDD); 1 female, Abkhazia, Bzybian Mt. Range, vicinities of Village Duripsh, 04.VI.1988, leg. V. Karmanian (cSIA); 1 male, Abkhazia, Gudauta Distr. near Village Khuap, 25.V–05.VIII.1986 (cSIA); 1 male, Abkhazia, Gudauta Distr., Khipsta Gorge, 580 m (cPNYu); 1 male, Abkhazia, Gudauta Distr., valley of River Khipsta, vicinities of Village Tvanaarkhu, karst crater with cave, beech, blackberry site, 425 m, N43°14', E040°39'9", 08.V–05.VII.2013, leg. I. Solodovnikov, S. Solodovnikova, S. Pavlyuchuk (cSIA); 1 male, 1 female, Abkhazia, Gudauta Distr., valley of River Khipsta, vicinities of Village Tvanaarkhu, valley of small brook, 380–425 m, hornbeam, beech, box forest site, N43°13', E040°39', 08.V–05.VII.2013, leg. I. Solodovnikov, S. Solodovnikova, S. Pavlyuchuk (cSIA, cPSM); 13 males, 17 females, Abkhazia, Gudauta Distr. near Village Khuap, 700 m, 11.IV–6.VI.2014, leg. D. Fominykh and A. Bondarenko (cFDD, cBAS).

#### Description.

The medium-sized form, males 32.5–32.5 (32.5) mm, females 32.5–32.6 (32.6) mm long (according to Kurnakov 1972 – 28–33 mm long). Underside black, dorsum normally with bright metallic lustre, violet, black-violet, black, rather seldom green, bronze, crimson, females matte. Body slender.

Head not inflated. Pronotum variable in shape, mainly transverse. PW/PL = 1.30–1.40 (1.35) in males and 1.27–1.28 (1.28) in females, with approximately same PW/PB as in the nominotypical subspecies: 1.27–1.32 (1.30) in males and 1.16–1.26 (1.21) in females, hind angles strongly protruding backwards and somewhat sidewards, pointed apically. Median groove distinct. Disk transversally rugose, rugosity gradually strengthening towards median groove. Elytra oblong-ovate, more elongate and somewhat convex in males, with quite inconspicuous depression in the middle in females. EL/EW = 1.77–1.80 (1.79) in males and 1.81–1.86 (1.83) in females. Elytral sculpture nearly identical in males and females, forming precise series of short links. The main morphometric measurements are presented in Table 2.

The endophallus differs from that of Carabus (Archiplectes) satyrus in the shape of its dorsal appendix, which is more elongate and possesses a more extended form, characteristic for populations dwelling in an interfluve of r. Kealasur and Bzyb.

#### Differential diagnosis.

Habitually resembles Carabus (Archiplectes) besleticus napraensis from which it differs in the more elongate and less ovate elytra, cordate pronotum with more elevated and sharper lateral borders, and stronger protruding hind angles. Elytral sculpture is more coarse, often with faintly developed tertiary interspaces, especially in females, body size on average larger. It also differs in habitat: Carabus (Archiplectes) besleticus duripshensis populates forest belts, rising up to the subalpine belt, while Carabus (Archiplectes) besleticus napraensis dwells exclusively at alpestrine and alpine zones. Morphometric characters are illustrated in Figures 1–2.

#### Distribution.

Populates southern slopes of Bzybian karstic figau at altitudes ranging from 380 to 1800 m, to the west from the River Khipsta.

#### Habitat.

Prefers beech and beech-chestnut forest sites, rich in ground litter on karstic landforms. Activity of imago proceeds from April until July. Carabus (Archiplectes) polychrous polychrous Rost, 1892, Carabus (Archiplectes) apollo tenebricosus, Carabus (Microplectes) argonautarum reischitzi, Carabus (Tribax) apschuanus apschuanus, Carabus (Tribax) constantinovi otcharensis, Carabus (Tribax) circassicus circassicus Ganglbauer, 1886 (natio *abasinus* Rost, 1893), Carabus (Megodontus) septemcarinatus, Carabus (Sphodristocarabus) armeniacus dvorschaki Mandl, 1975, and Carabus (Procerus) caucasicus colchicus occur together with this subspecies.

### Carabus (Archiplectes) besleticus napraensis

Taxon classificationAnimaliaColeopteraCarabidae

Belousov & Zamotajlov, 1993

Figs 1 , 2 , 7 , 25–27 , 62–65

Carabus (Archiplectes) satyrus napraensis Belousov & Zamotajlov, 1993: 53 (“Bzybian Mt Range, vicinity of Mt Chibzharga”); Bousquet et al. 2003: 132; Retezár 2008: 41.
Carabus (Tribax) satyrus napraensis : Deuve 2004: 275.

#### Comparative material examined.

50 paratype specimens were examined (4 specimens measured, 6 male genitalia preparations studied): 21 males, 29 females, Abkhazia, Gudauta Distr., Bzybian Mt. Range, vicinities of Mt. Chibzharga, 1900–2000 m, 20.VI–26.VII.1992, leg. I. Belousov, A. Zamotajlov, A. Miroshnikov (cFDD, cSIA, cZAM).

#### Description.

Small or medium-sized form, 22–31 mm long (see Belousov and Zamotajlov 1993). Underside black, dorsum normally with bright metallic lustre, green, bronze, less often dark blue, violet, black-violet, black, females matte to faintly nitidous. Body slender.

Head not inflated. Pronotum variable in shape, subcordate, transverse. PW/PL = 1.27–1.31 (1.29) in males and 1.48–1.49 (1.48) in females, PW/PB = 1.18–1.27 (1.22) in males and 1.20–1.22 (1.21) in females, hind angles strongly protruding backwards, somewhat pointed apically. Median groove distinct in females, microsculpture of pronotum fine, disk transversally or irregularly rugose, rugosity gradually strengthening towards median groove. Elytra oblong-ovate, more elongate and somewhat convex in males, with inconspicuous depression in the middle in females. EL/EW = 1.67–1.74 (1.70) in males and 1.70–1.81 (1.75) in females. Elytral sculpture distinctly heterodynamous, forming distinct series of short links in females and somewhat smoothed in males. The main morphometric measurements of studied populations see Table 2.

Shape of male genitalia is practically identical to other Carabus (Archiplectes) besleticus taxa. The endophallus differs from Carabus (Archiplectes) satyrus in the shape ofsaccellus, which is more elongate and more redundant, characteristic for populations dwelling in an interfluve of r. Kealasur and Bzyb. The drawing of the paratype aedeagus by Belousov and Zamotajlov (1993) shows different apical lamella with two characteristic tubercles on the sides.

#### Differential diagnosis.

Habitually resembles Carabus (Archiplectes) besleticus duripshensis from which it differs in a less elongate and more ovate, often ovoid, elytral shape, less cordate pronotum with fainter protruding hind angles, smooth elytral sculpture, and smaller average body size. This subspecies also differs in habitat – Carabus (Archiplectes) besleticus duripshensis populates forest belt, and less often an alpestrine belt of Mt. Chipshira, while *Carabus besleticus napraensis* dwells in alpestrine and alpine belts. Morphometric characters of this subspecies are illustrated in Figures 1–2.

#### Distribution.

As far as it is known, populates alpine and alpestrine belts of Bzybian karstic figau near mountains Napra and Chibzharga at 1900–2250 m.

#### Habitat.

Carabus (Archiplectes) satyrus napraensis prefers herb alpine and subalpine meadows. Activity of imago proceeds from May to August, beetles being active when snow cover starts melting. Carabus (Megodontus) septemcarinatus, Carabus (Procechenochilus) adangensis gusevi Zamotajlov & Koval, 1989, Carabus (Procrustes) clypeatus kurnakovi Kryzhanovskij, 1968, Carabus (Tribax) certus Reitter, 1896, and Carabus (Tribax) circassicus circassicus (natio *abasinus*) occur together with this subspecies.

### Carabus (Archiplectes) besleticus adzinbai

Taxon classificationAnimaliaColeopteraCarabidae

Retezár, 2013

Figs 4 , 7

Carabus (Archiplectes) satyrus adzinbai Retezár, 2013: 2

#### Comparative material examined.

15 specimens were examined (12 specimens measured, 1 male genitalia preparations studied): 1 male, 2 females, Abkhazia, Bzybian Mt. Range, N slope of Mt. Akibakhu (=Turetskaya shapka), 2000 m, alpine zone, 14.VI.-09.VIII.1986, leg. A. Koval (cZAM); 1 male, 10 females, Abkhazia, Bzybian Mt. Range, N slope of Mt. Akibakhu (=Turetskaya shapka), 2000 m, alpine zone, 7–10.VII.2010, leg. D. Fominykh, A. Bondarenko (cFDD, cSIA); 1 female, Abkhazia, Gudauta distr., S slope Bzybian Mt.R., NW Mt.Akugra, 43°18'N / 40°43'E, h = 2130–1150 m, alpine zone, 08.08.2014, leg. I. Solodovnikov, E. Tatun (cSIA).

#### Description.

Large form, males 31.0–32.0 mm, according to Retezár (2013) 32–34 mm long, females 32.0–35.8 mm, according to Retezár (2013) 33–37 mm long. Underside black, dorsum usually with bright metallic lustre, dark bronze, greenish-bronze, dark blue, reddish-bronze or black, pronotum often greenish; mandibles, palpi, antennae, and legs black. Habitus see Retezár, 2013.

Head normal, frons coarsely, neck moderately wrinkled. Pronotum subquadrate, broadest in anterior one third, PW/PL = 1.14–1.42 (1.25), PW/PB = 1.0–1.26 (1.14), lateral sides of pronotum slightly sinuated before hind angles, the latter strongly protruding backwards and sidewards, pointed apically. Median groove distinct, disk moderately, basal foveae coarsely rugose. Elytra oblong-ovate, broadest behind their middle, EL/EW = 1.61–1.77 (1.69) in males and 1.64–1.85 (1.72) 1.73–1.98 in females. Elytral sculpture somewhat identical in males and females, forming coarse and precise series of links, primary interspaces elevated stronger than secondary ones, regularly interrupted by large foveae. Table 2 lists the main morphometric measurements of one studied population.

The shape of the male genitalia is practically identical to that of the other Carabus (Archiplectes) besleticus taxa.

#### Differential diagnosis.

This form recently described by Retezár (2013), resembles habitually some populations of Carabus (Archiplectes) besleticus mtsaranus, but unlike them represents somewhat different combination of features. However, the significant and stable difference is not still ascertained, accumulation of the further material from the upper forest belt will apparently make possible unequivocal precision of its taxonomic relationships with other neighboring populations.

#### Distribution.

Populates the alpine belt of Mt. Khipsta, Mt. Akugra, and Mt. Akibakhu at an altitude of 2000–2300 m.

#### Habitat.

Prefers alpine herb meadows. Activity of imago proceeds from May to August, beetles being active at melting of snow cover. Carabus (Procechenochilus) gusevi, Carabus (Tribax) circassicus circassicus (natio *abasinus*), Carabus (Tribax) constantinovi otcharensis, Carabus (Tribax) agnatus, Carabus (Lipaster) stjervalli humbolti Faldermann, 1835, and Carabus (Pachycarabus) imitator katharinae Reitter, 1896 occur together with this subspecies.

### Carabus (Archiplectes) besleticus dsychvensis

Taxon classificationAnimaliaColeopteraCarabidae

Gottwald, 1985

Figs 1 , 2 , 7 , 28–30 , 74–77

Carabus (Archiplectes) juenthneri dsychvensis Gottwald, 1985: 304 (“Mt. Dsykhwa-Nordhang”); Retezár 2008: 39.
Carabus (Archiplectes) reitteri dsychvensis : Bousquet et al. 2003: 132.
Carabus (Tribax) juenthneri dsychvensis : Deuve 2004: 273.
Carabus (Archiplectes) satyrus dsychvensis : Retezár 2013: 4.

#### Comparative material examined.

278 specimens were examined (37 specimens measured, 12 male genitalia preparations studied): 151 males, 127 females, Abkhazia, Bzybian Mt. Range, SW slopes of Mt. Dzykhva, 2000–2300 m, alpine zone, N43°13', E041°08', 29.VI–03.VII.2013, leg. D. Fominykh (cFDD, cTAYu, cSAA, cSIA, cKVM).

#### Description.

Small form, males 23.3–26.5 (25.2) mm and females 23.3–26.5 (25.2) mm long. Underside black, dorsum normally with bright metallic lustre, green, bronze, less often dark violet or dark blue. Red femoral forms are also rather frequent. Body slender.

Head not inflated. Pronotum variable in shape, subcordate, transverse. PW/PL = 1.20–1.58 (1.43) in males and 1.30–1.62 (1.46) in females, PW/PB = 1.07–1.37 (1.22) in males and 1.03–1.30 (1.17) in females, hind angles protruding backwards, pointed apically. Median groove distinct in females, and inconspicuous and smoothed in males, disk with fine rugosity, gradually strengthening towards median groove. Elytra oblong-ovate, more elongate and somewhat convex in males, with inconspicuous to missing middle depression in the females. EL/EW = 1.56–1.85 (1.68) in males and 1.50–1.79 (1.66) in females. Elytral sculpture smooth in males and precise, forming distinct series of short links in females. The main morphometric measurements are presented in Table 2.

Apical lamella of aedeagus of transitional between Carabus (Archiplectes) besleticus and Carabus (Archiplectes) pseudopshuensis shape, possessing a sharp hollow on the right between apex and tubercle. Also sharply differs in this respect from Carabus (Archiplectes) juenthneri (previously considered as its subspecies). The endophallus differs from Carabus (Archiplectes) satyrus in the shape of the dorsal appendix, which is more elongate and possesses a more extended form, characteristic for populations dwelling in an interfluve of the r. Kealasur and Bzyb.

#### Differential diagnosis.

Habitually resembles Carabus (Archiplectes) juenthneri adsypschi Gottwald, 1985, from which differs in more elongate and more ovate elytra, hind angles of pronotum more strongly protruding, elytral sculpture more coarse, and larger average body size. Also easily distinguishable by male genitalia. The apical lamella of this form combines some features of both Carabus (Archiplectes) besleticus and Carabus (Archiplectes) pseudopshuensis. The shape of the saccellus and aggonoporius seems, however, to be closer to Carabus (Archiplectes) besleticus. The study of the further high-mountain populations of *Archiplectes* from adjacent woodless alpine massifs is required for correct interpretation of its taxonomic status within the species-complex in question. Morphometric characters are illustrated in Figures 1–2.

#### Distribution.

Populates the alpine belt of Mt. Dzykhva at 2000–2300 m altitude.

#### Habitat.

Prefers alpine herb meadows. Activity of imago proceeds from May to August, beetles being active at melting of snow cover. Carabus (Procechenochilus) adangensis, Carabus (Tribax) circassicus circassicus (natio *tshchaltensis* Novotný & Voříšek, 1988), Carabus (Tribax) constantinovi otcharensis, Carabus (Tribax) agnatus pseudoagnatus Novotný & Voříšek, 1988, and Carabus (Pachycarabus) imitator katharinae Reitter, 1896 occur together with this subspecies.

### Carabus (Archiplectes) besleticus resheviensis

Taxon classificationAnimaliaColeopteraCarabidae

Solodovnikov, Zamotajlov & Fominykh subsp. n.

Figs 1 , 2 , 3 , 4 , 5 , 6 , 7 , 31–33 , 66–73

#### Type material.

Holotype, a male: Abkhazia, N Slopes of Bzybian Mt. Range, right bank of River Reshevie (= Reshava) (left tributary of River Bzyb), 700–1000 m, 13.V–02.VII.2010, leg. D. Fominykh, I. Solodovnikov (ZISP). 577 paratypes: 34 males, 48 females, Abkhazia, N Slopes of Bzybian Mt. Range, right bank of River Reshevie (left tributary of River Bzyb), 700-1000 m, 13.V–02.VII.2010, leg. D. Fominykh, I. Solodovnikov (cZAM, cFDD, cSIA); 234 males, 261 females, same locality, 04.V-20.VII.2012, leg D. Fominykh, A. Safronov (cZAM, cFDD, cSIA, cSAA, cBAS, cTAYu); 3 females, Abkhazia, Bzybian Mt. Range, right bank of River Reshevie (left tributary of River Bzyb), 670–720 m, 13–14.V.2010, leg. D. Fominykh, I. Solodovnikov (cPRYu); 5 males, 3 females, Abkhazia, Sukhum Distr., left bank of River Bzyb, right bank of River Reshevie, beech, maple, fern forest site, 670–720 m, 13–14.V.2010, leg. I. Solodovnikov (cSIA); 5 males, 10 females, same locality, 13.V–02.VII.2010 leg. I. Solodovnikov, D. Fominykh (cSIA, cPIG, cKVM); 1 male, labeled “Cauc. occ. Abchasia Pskhu 1910, *Plectes protensus* Schaum” (ZISP); 1 female, labeled “West Caucasus, Pskhu, VII.1913, Satunin” (ZISP). Holotype and 57 paratype specimens measured, 30 male genitalia preparations studied.

#### Description.

Large form, body size 34.5–43.0 mm, males 34.5–39.0 (37.6) mm, females 37.2–43.0 (40.6) mm long. Underside black, dorsum bright bronze, red-gold, green, violet, dark blue, black, with metallic lustre in males and matte in females. Appendages black, rarely the femora are red and basal antennomeres reddish-brown (approx. 5 % of individuals). Body usually monochromatic dorsally, less frequently bicolored (head and pronotum being usually greenish, the elytra bronze or reddish-bronze). Body massive and rather high (Figs 66–73).

Head huge, somewhat inflated. Pronotum transverse to subquadrate, hind angles protruding backwards and in some individuals sidewards. PW/PL = 1,18–1,55 (1,37) in males and 1,29–1,67 (1,45) in females. Elytra faintly elongate, ovate; more elongate, sometimes with depression in the middle in females, EL/EW = 1,52–1,92 (1,72); generally more ovate, convex, and smoothed in males, EL/EW = 1,42–1,78 (1,59). Elytral sculpture precise to homodynamous and confused, with hardly any distinguishable primary interspaces in males. The main morphometric measurements are presented in Table 2. Male genitalia practically identical to other taxa of Carabus (Archiplectes) besleticus.

#### Differential diagnosis.

Carabus (Archiplectes) besleticus resheviensis resembles habitually a population of Carabus (Archiplectes) besleticus besleticus, inhabiting valley of River Basla (city of Sukhum vicinities), but differs in the following characters. Pronotum with a rather shallow median depression, being only poorly marked in males; disk impressed; hind angles huge, appreciably larger, lateral sides of pronotum strongly sinuated before hind angles; elytra broader in males, EL/EW = 1.59, while 1.75 in Carabus (Archiplectes) besleticus besleticus and 1.61–1.75 in Carabus (Archiplectes) besleticus mtsaranus. The new subspecies is the closest geographically to Carabus (Archiplectes) besleticus mtsaranus, populating southern slopes of Bzybian Mt. Range, but differs in having a larger body size, more ovate elytra, smooth elytral sculpture, more transverse pronotum with weaker protruding hind angles, and general dorsal coloration (PW/PL = 1.14–1.48 (1.35) in males and 1.26–1.56 (1.41) in females of Carabus (Archiplectes) besleticus mtsaranus); furthermore, the disk of the pronotum is more convex than in the new subspecies. The new subspecies differs from the recently described Carabus (Archiplectes) besleticus adzinbai first of all in having a larger body size, more transverse and faintly cordate pronotum with more narrow base; Carabus (Archiplectes) besleticus adzinbai possesses subquadrate pronotum with stronger protruding sidewards hind angles, PW/PL = 1.14–1.42 (1.25), PW/PB = 1.00–1.26 (1.14); elytra of Carabus (Archiplectes) besleticus resheviensis are more ovate and shorter both in males and females.

The bulk of studied individuals of Carabus (Archiplectes) besleticus resheviensis (males) differ from the other subspecies, namely Carabus (Archiplectes) besleticus besleticus, Carabus (Archiplectes) besleticus mtsaranus, Carabus (Archiplectes) besleticus duripshensis, and Carabus (Archiplectes) besleticus adzinbai in their elytral sculpture: tertiary interspaces are elevated almost as secondary ones, forming a smooth sculpture, primary interspaces interrupted more frequently. In the other subspecies tertiary interspaces are normally hardly raised. Morphometric characters are illustrated in Figures 1–6.

#### Distribution.

Populates boreal slopes of Bzybian Mt. Range on the right bank of River Reshevie (=Reshava) at 700–1000 m. Occurs sympatrically with Carabus (Archiplectes) pseudopshuensis, the population density of the latter being somewhat higher. Occurrence of this subspecies at the left bank of River Reshevie and downstream River Bzyb seems also possible, but collected material does not confirm this possibility at present.

#### Habitat.

Prefers slightly sloping beech and fir-beech forest sites rich in ground litter on karstic landforms. Activity of imago proceeds from April to July. Carabus (Archiplectes) pseudopshuensis, Carabus (Tribax) apschuanus apschuanus, Carabus (Tribax) constantinovi otcharensis, Carabus (Tribax) circassicus circassicus (natio *abasinus*), Carabus (Microplectes) argonautarum reischitzi, Carabus (Megodontus) septemcarinatus, Carabus (Sphodristocarabus) armeniacus dvorschaki, and Carabus (Procerus) caucasicus colchicus occur together with this subspecies.

#### Subspecific epithet.

The subspecific epithet refers to the name of the River Reshevie, the type locality of subspecies.

### Carabus (Archiplectes) pseudopshuensis

Taxon classificationAnimaliaColeopteraCarabidae

Zamotajlov, 1991

Figs 5 , 6 , 7 , 12–15 , 78–89

Carabus (Archiplectes) satyrus pseudopshuensis Zamotajlov, 1991: 36 (“Environs of Pskhu”); Bousquet et al. 2003: 132; Retezár 2008: 41;
Carabus (Tribax) satyrus pseudopshuensis : Deuve 2004: 275.
Carabus (Archiplectes) pseudopshuensis : Fominykh and Zamotajlov 2012: 444; Retezár 2013: 4.

#### Comparative material examined.

3351 specimens were examined (135 specimens measured, 60 male genitalia preparations studied): 2 males, 4 females, Abkhazia, Sukhum Distr., left bank of River Aguripsta near outfall of River Belaya, beech forest site, 800–1000 m, 11.V–27.VI.2010, leg. D. Fominykh, I. Solodovnikov (cZAM, cFDD, cSIA); 5 males, 5 females, Abkhazia, Sukhum Distr., left bank of River Aguripsta near Mt. Svyataya, 800 m, 10.V–28.VI.2010, leg. D. Fominykh, I. Solodovnikov (cZAM, cFDD); 4 males, 2 females, Abkhazia, Sukhum Distr., vicinities of Village Pskhu, S slope of Mt. Svyataya, beech forest site, 650–700 m, 10.V–28.VI.2010, leg. I. Solodovnikov, D. Fominykh, (cSIA); 21 males, 25 females, Abkhazia, Sukhum Distr., SE of Village Pskhu, hamlet Bitaga, Mt. Bzybskaya, 550–650 m, beech forest site, 12.V–30.VI.2010, leg. I. Solodovnikov, D. Fominykh, (cSIA); 547 males, 763 females, Abkhazia, Sukhum Distr., left bank of River Aguripsta near Village Pskhu, W slopes of Mt. Chibiskha, 700 m, 25.IV–04.VII.2013, leg. D. Fominykh (cZAM, cFDD, cSAA, cBAS); 48 males, 62 females, Abkhazia, Sukhum Distr., right bank of River Bzyb, S slopes of Mt. Chibiskha, 550 m, 25.IV–04.VII.2013, leg. D. Fominykh (cZAM, cFDD); 5 males, 4 females, Abkhazia, Sukhum Distr., right bank of River Bzyb, S slopes of Mt. Chibiskha upstream hamlet Bitaga, karst, beech forest site, 600–620 m, N43°21'29.36" / E040°49'58.89", 25.IV–04.VII.2013, leg. D. Fominykh (cSIA); 6 males, 6 females, Abkhazia, Sukhum Distr., right bank of River Baul near Village Sanchara, 1030 m, N43°24,9', E040°51,4', VII.2010, leg. I. Retezár (cZAM, cFDD); 2 males, 2 females, Abkhazia, Sukhum Distr., vicinities of Village Sanchara, River Baul, beech forest site, 20.VII.1991, leg. M.N. Maksimenkov (cSIA); 2 females, same locality, 800 m, 10.VIII.1989, leg. M.N. Maksimenkov (cSIA); 2 males, Village Sanchara, VI.1991, leg. M.N. Maksimenkov (cSIA); 1 male, Abkhazia, Sukhum Distr., Pskhu, 10.IX.1991, local collector (cSIA); 3 males, 4 females, Abkhazia, Sukhum Distr., Bzybian Mt. Range, upper reaches of RRiver West Gumista, Dou Pass, 1300 m, 30.IV–19.VII.2012, leg. D. Fominykh (cFDD); 10 males, 13 females, Abkhazia, Sukhum Distr., N slopes of Bzybian Mt. Range near Bzyb valley, 1000 m, 30.IV.2012, leg. D. Fominykh (cZAM, cFDD, cSAA); 40 males, 30 females, Abkhazia, Sukhum Distr., N slopes of Bzybian Mt. Range near Bzyb valley, 800 m, 30.IV–19.VII.2012, leg. D. Fominykh (cZAM, cFDD, cSIA, cSAA); 351 males, 449 females, Abkhazia, Sukhum Distr., N slopes of Bzybian Mt. Range, right bank of River Reshevie, 700–1000 m, 1.V–20.VII.2012, leg. D. Fominykh (cZAM, cFDD, cSAA, cBAS); 2 males, 5 females, Abkhazia, Sukhum Distr., left bank of River Bzyb, right bank of River Reshevie, beech, maple, fern forest site, 670–720 m, 13–14.V.2010, leg. I. Solodovnikov (cSIA); 9 males, 18 females, same locality, 13.V-02.VII.2010, leg. I. Solodovnikov, D. Fominykh (cSIA, cPIG, cKVM); 14 males, 16 females, same locality, 04.V–20.VII.2012, leg. D. Fominykh, A. Safronov (cSIA); 386 males, 516 females, Abkhazia, Sukhum Distr., N Slopes of Bzybian Mt. Range near Village Serebryanyi, 500–600 m, 25.IV–05.VII.2013, leg. D. Fominykh (cZAM, cFDD); 5 males, 8 females, Abkhazia, Sukhum Distr., vicinities of Village Pskhu, hamlet Serebryanyi, left bank of River Bzyb, karst, beech forest site, 600–620 m, N43°21'52.90" / E040°48'52.90", 25.IV–05.VII.2013, leg. D. Fominykh (cSIA).

#### Description.

Medium-sized form, length 26.5–34.0 mm, males 28.0–32.0 mm, females 28.5–34.0 mm long. Underside black, dorsum normally with bright metallic lustre, green, dark blue, bronze, crimson, violet, less often black, with transitional color forms in males, dichromatic and trichromatic individuals being also known, females matte. Body slender.

Head not inflated. Pronotum variable in shape, from transverse to subquadrate. PW/PL of individuals from the right bank of River Bzyb hardly depends upon altitude in males and constitute constantly 1.32–1.39, in females, upon decrease of altitude, PW/PL varies from 1.38 at 700 m to 1.48 at 1100 m. Hind angles slightly protruding backwards. In the left-bank populations, pronotum of nearly the same proportions or less transverse, particularly in females. PW/PL varies there in males of three studied populations from 1.26 to 1.39, and in females – from 1.34 to 1.42, reaching extreme value of 1.23 in the population, inhabiting environs of the hamlet Serebryanyi. Elytra oblong-ovate in the right-bank populations, also vary in shape from distinctly elongate to almost ovate. EL/EW gradually varies in males depending upon altitude decrease from 1.89 to 1.69, in females such correlations are not obviously revealed and irregularly varies from 1.69 to 1.76. Elytral sculpture varies from almost smooth to coarsely granular, with strongly pronounced punctation in the middle interspaces. EL/EW is more or less stable in the left-bank populations and measures 1.66–1.70 in males and 1.60–1.75 in females. Elytral sculpture granular, interspaces regular, nearly straight, some male specimens possess somewhat confused elytral sculpture. The main morphometric measurements are presented in Table 1.

The structure of endophallus testifies to the assignment of this form to the *reitteri*-group. It is easily distinguishable from Carabus (Archiplectes) satyrus, Carabus (Archiplectes) besleticus, and Carabus (Archiplectes) juenthneri by the shape of the dorsal appendix, which is less elongate and possesses a more rectangular form.

**Figures 78–81.**
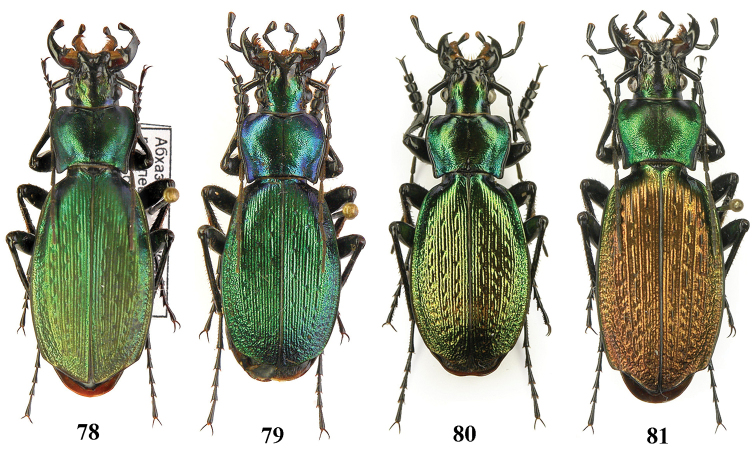
Dorsal habitus, Carabus (Archiplectes) pseudopshuensis. **78–79** Abkhazia, Village Pskhu env **80–81** Abkhazia, N slopes of Bzybian Mt. Range, Village Serebryanyi env.

**Figures 82–89.**
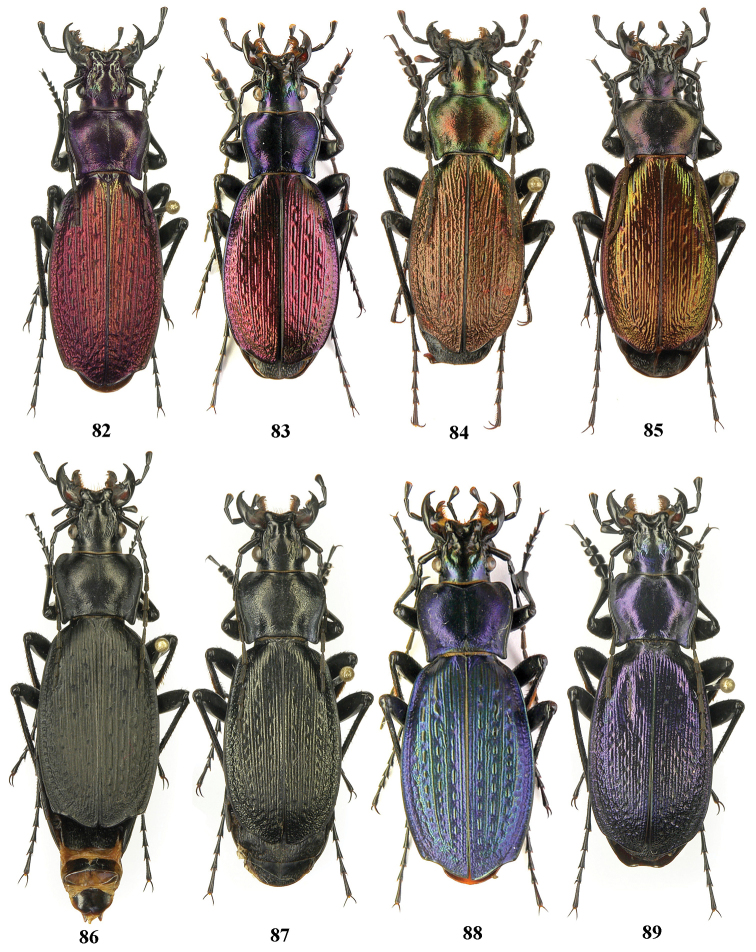
Dorsal habitus, Carabus (Archiplectes) pseudopshuensis. **82–83, 88–89** Abkhazia, N slopes of Bzybian Mt. Range, Village Serebryanyi env **84** Abkhazia, Village Sanchara env **85** Abkhazia, Village Pskhu env., Mt. Svyataya **86–87** Abkhazia, Village Pskhu env.

#### Differential diagnosis.

Strongly resembles Carabus (Archiplectes) juenthneri juenthneri, which occurs sympatrically in some localities on the right bank of River Bzyb; however, it differs in smaller body size, more elongate and ovate body, and also in the structure of the male genitalia. Upstream, in the valley of River Aguripsta, Carabus (Archiplectes) juenthneri juenthneri completely substitutes this taxon. On the left bank of River Bzyb it also occurs sympatrically with Carabus (Archiplectes) besleticus resheviensis from which it differs in considerably smaller body size and different male genitalia. The authors have succeeded in collecting in such localities few transitional individuals, apparently being hybrid forms. Morphometric characters are illustrated in Figures 5–6.

#### Distribution.

Populates the left bank of the River Aguripsta from the confluence of River Belaya (its left tributary) up to its outfall, and also right board of River Bzyb, upstream confluence of River Aguripsta, also occurs at northern slopes of Bzybian Mt. Range, reaching its watershed at 1300 m, where dwells together with Carabus (Archiplectes) apollo tenebricosus, however it has not been found at its southern slopes, where apparently it is completely substituted by the latter.

#### Habitat.

Inhabits mainly beech and fir-beech mixed forests, developed on rich ground litter, and also petrous forest taluses at karstic landforms. The population density is rather high in its known geographic range, except for the middle flow of River Aguripsta (natural border of its distribution area), the maximum density of imago is recorded for the altitude interval of 600–900 m. Activity of imago proceeds from April to July, solitary individuals are sometimes observed at the end of summer or the middle of September. The following forms of *Carabus* occur sympatrically on the left bank of River Bzyb: Carabus (Archiplectes) besleticus resheviensis, Carabus (Archiplectes) apollo tenebricosus, Carabus (Tribax) apschuanus apschuanus, Carabus (Tribax) constantinovi otcharensis, Carabus (Tribax) circassicus circassicus (natio *abasinus*), Carabus (Microplectes) argonautarum reischitzi, Carabus (Megodontus) septemcarinatus, Carabus (Eucarabus) cumanus Fischer von Waldheim, 1823, Carabus (Sphodristocarabus) armeniacus dvorschaki, and Carabus (Procerus) caucasicus colchicus; on the right bank of River Bzyb: Carabus (Archiplectes) juenthneri juenthneri, Carabus (Tribax) apschuanus schoeni Novotný & Voříšek, 1988, Carabus (Tribax) constantinovi otcharensis, Carabus (Tribax) circassicus circassicus (natio *abasinus*), Carabus (Microplectes) argonautarum reischitzi, Carabus (Megodontus) septemcarinatus, Carabus (Eucarabus) cumanus, Carabus (Pachycarabus) imitator katharinae Reitter, 1896, Carabus (Sphodristocarabus) armeniacus janthinus Ganglbauer, 1887, and Carabus (Procerus) caucasicus colchicus.

## Supplementary Material

XML Treatment for Carabus (Archiplectes) satyrus

XML Treatment for Carabus (Archiplectes) besleticus

XML Treatment for Carabus (Archiplectes) besleticus besleticus

XML Treatment for Carabus (Archiplectes) besleticus mtsaranus

XML Treatment for Carabus (Archiplectes) besleticus duripshensis

XML Treatment for Carabus (Archiplectes) besleticus napraensis

XML Treatment for Carabus (Archiplectes) besleticus adzinbai

XML Treatment for Carabus (Archiplectes) besleticus dsychvensis

XML Treatment for Carabus (Archiplectes) besleticus resheviensis

XML Treatment for Carabus (Archiplectes) pseudopshuensis
